# A bi-specific lectin from the mushroom *Boletopsis grisea* and its application in glycoanalytical workflows

**DOI:** 10.1038/s41598-020-80488-7

**Published:** 2021-01-08

**Authors:** Mehul B. Ganatra, Vladimir Potapov, Saulius Vainauskas, Anthony Z. Francis, Colleen M. McClung, Cristian I. Ruse, Jennifer L. Ong, Christopher H. Taron

**Affiliations:** New England Biolabs, Inc, 240 County Road, Ipswich, MA 01938 USA

**Keywords:** Proteomics, Glycosylation, Fungal biology

## Abstract

The BLL lectin from the edible Japanese “Kurokawa” mushroom (*Boletopsis leucomelaena*) was previously reported to bind to N*-*glycans harboring terminal *N-*acetylglucosamine (GlcNAc) and to induce apoptosis in a leukemia cell line. However, its gene has not been reported. In this study, we used a transcriptomics-based workflow to identify a full-length transcript of a BLL functional ortholog (termed BGL) from *Boletopsis grisea*, a close North American relative of *B. leucomelaena*. The deduced amino acid sequence of BGL was an obvious member of fungal fruit body lectin family (Pfam PF07367), a highly conserved group of mushroom lectins with a preference for binding O-glycans harboring the Thomsen–Friedenreich antigen (TF-antigen; Galβ1,3GalNAc-α-) and having two ligand binding sites. Functional characterization of recombinant BGL using glycan microarray analysis and surface plasmon resonance confirmed its ability to bind both the TF-antigen and β-GlcNAc-terminated N*-*glycans. Structure-guided mutagenesis of BGL’s two ligand binding clefts showed that one site is responsible for binding TF-antigen structures associated with O-glycans, whereas the second site specifically recognizes N-glycans with terminal β-GlcNAc. Additionally, the two sites show no evidence of allosteric communication. Finally, mutant BGL proteins having single functional bindings site were used to enrich GlcNAc-capped N-glycans or mucin type O-glycopeptides from complex samples in glycomics and glycoproteomics analytical workflows.

## Introduction

Lectins are non-catalytic proteins that reversibly bind to sugars. Individual lectins typically bind their ligands with a high degree of stereochemical selectivity. They are found in all domains of biology but have been most highly studied in plants and animals. Lectins play a vital role in many important biological processes including cell adhesion, cell signaling, glycoprotein folding, and protein trafficking. They also enable many pathogenic bacteria and viruses to bind to their cellular targets^1,2^. In addition to their importance in biology, lectins are also important tools in biotechnology and diagnostics^3,4^. Dozens of lectin specificities are available from various commercial suppliers, and lectins are used in research applications including histology, glycan immunoassays, glycan profiling and glycoprotein/glycopeptide separations.

In recent years, there has been increasing research interest in lectins isolated from fungi, in particular, mushrooms. Mushrooms abundantly produce lectins as storage proteins, and they are thought to provide a mechanism for defense against predation by insects^5^. Currently, over 100 different mushroom lectins have been described^6,7^. Interestingly, many mushroom lectins possess compelling bioactivities such as suppression of cancer cell proliferation and tumor growth, induction of lymphocyte mitogenesis, suppression of B and T cell activation, activation of macrophages and inhibition of tobacco mosaic virus and HIV reverse transcriptase^6–8^. Thus, mushrooms represent a compelling source for discovery of lectins with important bioactivities and novel specificities.

The BLL lectin (also termed KL-15) from the Japanese edible “Kurokawa” mushroom *Boletopsis leucomelaena* is among many reported mushroom lectins with bioactive properties. Isolation of BLL was first reported in a study from Japan in 2002^9^. In that study, purified BLL was shown to inhibit proliferation of human monoblastic leukemia U937 cells and induce apoptosis. Subsequent studies examined the binding specificity of BLL using frontal affinity chromatography and a panel of fluorescently labeled glycans^10,11^, and showed a preference of BLL to bind N*-*glycans with exposed terminal *N-*acetylglucosamine (GlcNAc) residues. We became interested in BLL’s specificity for potential use in enrichment of fluorescently labeled N*-*glycans having terminal GlcNAc using liquid chromatography (LC) coupled to fluorescence detection (FLR) and mass spectrometry (MS)^12^. However, obtaining BLL for use in glycan profiling was not feasible because *B. leucomelaena* mushrooms are uncommon in North America^13^. Additionally, the gene and full protein sequence of BLL had not been previously reported.

In this study, we report the use of a transcriptomics-based workflow to identify the full-length transcript sequence of a functional BLL ortholog from *B. grisea* (termed BGL), a closely related North American *Boletopsis* species^13^. The deduced BGL protein sequence was highly homologous to some members of a family of fungal fruit body lectins (Pfam PF07367) that are well-known for their ability to bind to O-glycans bearing the Thomsen-nouveau antigen (Tn antigen, GalNAc-**α**-O-Ser/Thr) or Thomsen–Friedenreich antigen (TF-antigen, Gal**β**1,3GalNAc-**α**-O-Ser/Thr), but not N*-*glycans with terminal GlcNAc. Crystal structures of these fruit body lectins indicate the presence of two monosaccharide-binding sites^14,15^, but the oligosaccharide binding specificity of each site has not been individually explored. Thus, we used a combination of structure-guided mutagenesis, glycan microarray binding, and surface plasmon resonance to further define the function and specificity of both ligand-binding sites. Finally, we show the utility of recombinant BGL (rBGL) and engineered highly specific mutant rBGL proteins in oligosaccharide enrichment schemes within common glycomics and glycoproteomics workflows.

## Results

### Mushroom speciation

A putative Kurokawa mushroom was obtained via a North American commercial mushroom supplier. We confirmed the species of the acquired mushroom by both morphological and molecular analyses. The mushroom had a dark gray and brown cap and produced a spore print of small elliptical bumpy spores, a morphological signature of the *Boletopsis* genus^16^ (Supplement Fig. S1A–C). Molecular speciation was performed through amplification and sequencing of the fungal 5.8S rRNA gene and flanking internal transcribed spacer (ITS) regions (ITS1 and ITS2) as described in the “Material and Methods” (Supplement Fig. S1 D). Nucleotide sequences of the amplified ITS1 and ITS2 regions (GenBank KT315925) were identical to those previously reported for *B. grisea* (GenBank EF457902) and had only minor differences to those of *B. leucomelaena* (GenBank DQ408771) (Supplement Fig. S2). Thus, we concluded that the mushroom obtained for this study was *B. grisea*, the most common North American *Boletopsis* species and a close relative of *B. leucomelaena*.

### Identification of a *B. grisea* lectin cDNA

**Figure 1:**
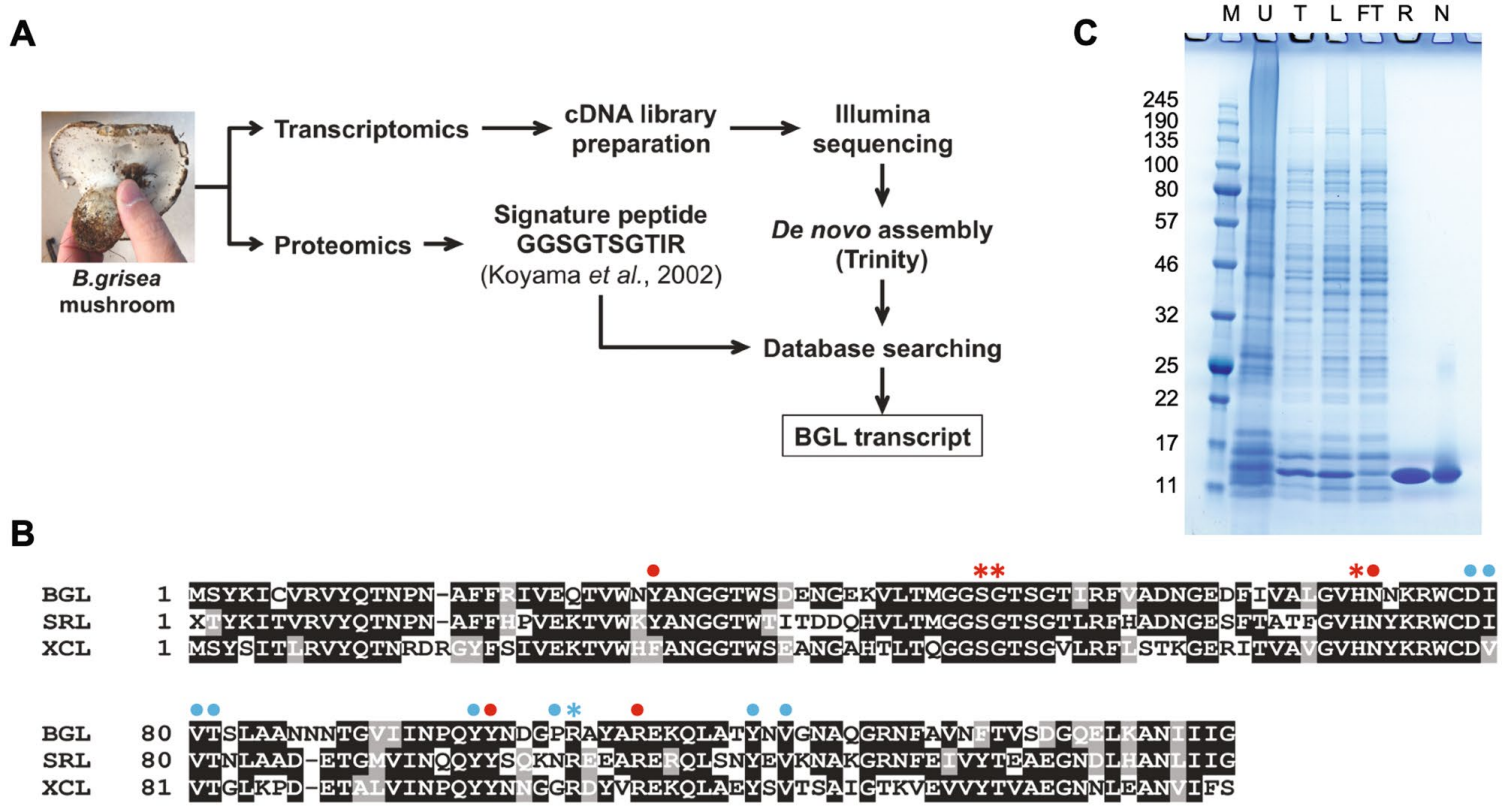
Identification and expression of a BGL transcript. (**A**) A combined transcriptomics and proteomics workflows were used to identify a BGL transcript. Total RNA was isolated and a directional cDNA library was prepared for RNA-Seq. De novo assembly without a reference genome was performed using Trinity software^17^. The signature peptide sequence (GGSGTSGTIR) was previously generated by CNBr and N-terminal sequencing^9^. BLASTX using GGSGTSGTIR as the query was used to probe the assembled *B. grisea* transcriptome to identify a full-length BGL transcript. (**B**) The deduced peptide sequence of BGL (143 a.a.) was aligned with those of *S. rolfsii* lectin (SRL) and *X. chrysenteron* lectin (XCL) using T-Coffee^39^ and BoxShade^40^. Identical residues are shaded black, and similar residues are shaded gray. Amino acids that comprise the primary and secondary ligand binding sites of SRL are shown with red or blue symbols (dots and asterisks), respectively (see also Supplement Fig. S4). Four amino acids (asterisks) were individually mutated to disrupt binding in the respective pockets. (**C**) *E. coli*-expressed and purified recombinant BGL was visualized by separation via 10–20% Tris–glycine SDS-PAGE and SimplyBlue staining. M: broad range molecular weight protein standard (kDa, New England Biolabs catalog #P7712S); U: uninduced lysate; T: total induced lysate; L: soluble lysate; FT: column flow-through; R: purified recombinant BGL; N: purified native BGL. The uncropped gel image is shown in Supplement Fig. S10.

To identify a candidate cDNA encoding a *B. grisea* ortholog of the *B. leucomelaena* GlcNAc-binding BLL protein, an Illumina transcript library was created from isolated *B. grisea* mRNA and subjected to deep sequencing. Raw sequence data (SRA SRR090126) was analyzed using Trinity, a software package developed to assemble transcript sequences in the absence of a reference genome^17^. Trinity de novo assembly yielded 9188 putative transcript sequences that were deposited in GenBank (TSA GEZR00000000). The program BLASTX was used to translate these sequences and compare the translations to a previously reported peptide (GGSGTSGTIR) that was obtained from amino-terminal sequencing of a 13 kDa CNBr digestion product of BLL^9^. A single 1989 nucleotide transcript sequence (GenBank KT315924) that harbored a 143 amino acid ORF (referred to as BGL) containing the GGSGTSGTIR sequence was identified (Fig. 1A).

The deduced BGL amino acid sequence was further analyzed by BLASTP analysis against protein sequences in GenBank. BGL was an obvious member of a conserved family of fungal fruit body lectins (Pfam PF07367). The protein structure of this lectin family was first defined by crystallization of the XCL lectin (GenBank AAL73235) from *Xerocomus chrysenteron* (now *Xerocomellus chrysenteron*)^18^ and the SRL lectin from *Sclerotium rolfsii* (GenBank ACN89784)^14^. BGL showed 57% and 67% sequence identity to XCL and SRL, respectively, and was of similar length (Fig. 1B). Interestingly, BGL showed little sequence level homology with the larger GlcNAc-binding PVL lectin from the mushroom *Psathyrella velutina* (GenBank DQ232759)^19^.

BGL over-production in *E. coli* was driven by the T7 promoter in pET21a(+). The expressed protein was soluble and clearly visible in *E. coli* cell lysates following induction. Recombinant BGL (rBGL) readily bound to GlcNAc agarose directly from *E. coli* lysates indicating that it was functional. The protein was purified to homogeneity by passage over GlcNAc agarose resin as described in the “Materials and methods” (Fig. 1C), and 420 mg of pure rBGL was obtained from 6 L of induced *E. coli* culture. The purified protein migrated as a single ~ 15 kDa band via reducing SDS-PAGE consistent with its calculated molecular weight of 15.75 kDa (Fig. 1C). Additionally, electrospray ionization mass spectrometry of rBGL yielded a molecular mass of 15.62 kDa, corresponding to the protein lacking an N-terminal methionine (Supplement Fig. S3).

### Glycan microarray analysis of rBGL

**Figure 2:**
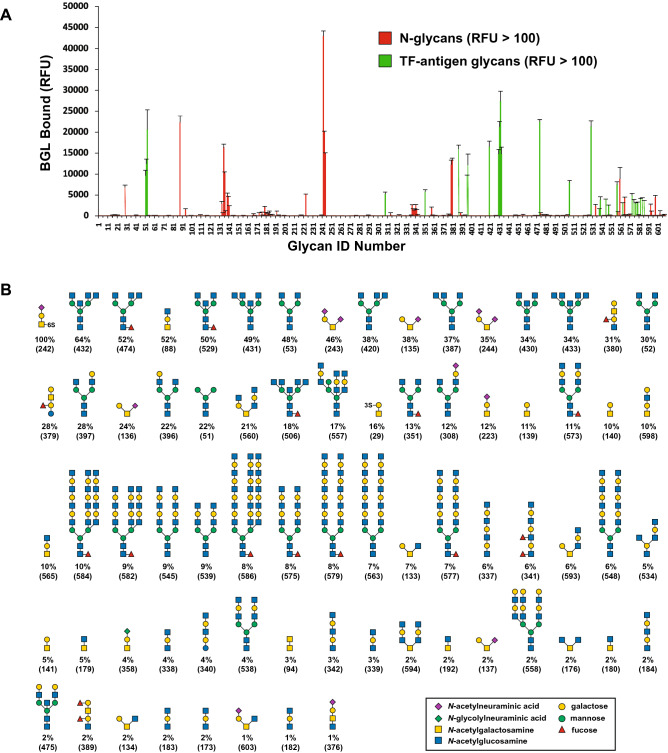
Oligosaccharide binding specificity of rBGL using a mammalian glycan array. Fluorescent dye-labeled rBGL (200 μg/mL) was used for glycan array screening at the Protein–Glycan Interaction Core H of the Consortium for Functional Glycomics (CFG). (**A**) The specificity of rBGL was determined by testing its ability to bind to a printed array (version 5.2) consisting of 609 mammalian glycans. Two distinct classes of glycan were bound; N*-*glycans having terminal GlcNAc (green bars) and glycans predominantly containing the TF-antigen disaccharide Galβ1,3GalNAc (red bars). (**B**) The structures of oligosaccharides bound by rBGL are shown in decreasing order of ligand binding efficiency down to 1% binding relative to the strongest signal (see Supplementary Table 1 for raw array data). The CFG array’s Glycan ID numbers for each structure are shown in parentheses. Structures appearing more than once reflect identical glycans in the array that possess different chemical linkers. *RFU* relative fluorescence units.

Recombinant BGL was assessed for its ability to bind to a variety of mammalian glycans. Fluorescently labeled rBGL was used to probe the Consortium for Functional Glycomics’ Protein–Glycan Interaction Core microarray (version 5.2) consisting of 609 natural and synthetic mammalian glycans. rBGL bound to 72 glycans with an average relative fluorescence (RFU) greater than 430 (1% of the strongest observed signal) (Fig. 2A,B). Raw data for these experiments is provided in Supplementary Table 1. Glycans bound by rBGL predominantly fell into two distinct structural classes.

Recombinant BGL bound to a group of small glycans containing a Galβ1,3GalNAc-α- epitope (Fig. 2A). This disaccharide motif is common to O-glycan Core 1 and 2 species, and to ganglioside-series glycosphingolipids. The strongest binding ligand was a sialylated and sulfated trisaccharide containing Galβ1,3GalNAc-α- (Fig. 2B, Glycan ID 242). The same trisaccharide lacking 6-sulfate bound only 12% as efficiently (Glycan ID 223). Of 41 glycans possessing Galβ1,3GalNAc-α- in the array, 23 were bound by BGL (Fig. 2B, Supplementary Table 1). Additionally, Galβ1,3GalNAc-β- (Glycan ID 142) was not bound by rBGL suggesting that the alpha anomer of GalNAc is imperative for binding. Small GalNAc-containing O-glycan structures comprising Core 3 (GlcNAcβ1,3GalNAc-α-), Core 4 (GlcNAcβ1,3[GlcNAcβ1,6]GalNAc-α-), and Core 6 (GlcNAcβ1,6GalNAc-α-) were also bound by rBGL, albeit more weakly than Galβ1,3GalNAc-α-.

The second class of glycan recognized by rBGL was N-glycans bearing at least one terminal GlcNAc residue capping an outer arm. Of 136 N*-*glycans in the array, rBGL bound with varying degrees of efficiency to only 31. This suggests that rBGL does not associate with GlcNAc in the context of the chitibiose core that is common to all N-glycans. The array contains 28 N-glycans having terminal β-GlcNAc residues in an outer arm, and all 28 were recognized by rBGL (Fig. 2B, Supplementary Table 1). Of these, the strongest binding was to structures having one or more terminal GlcNAcβMan epitopes. Binding was also observed to a paucimannose N-glycan (Fig. 2B; Glycan ID 51) that lacks terminal GlcNAc. The terminal α1,6 and α1,3 mannoses in this structure are the sugars to which β-linked GlcNAc becomes attached in complex-type N-glycans. If GlcNAcβ1,2Man were a preferred epitope for rBGL, then it is plausible that in the absence of GlcNAc, rBGL might more weakly interact with only the α1,6 or α1,3 mannose residues in this structure.

Additionally, N-glycan structures bearing terminal β-GlcNAc in the context of outer arm *N-*acetyllactosamine (LacNAc; Galβ1,4GlcNAc) repeating disaccharides were also recognized, albeit more weakly. The strongest binding N-glycans of this class had a single LacNAc unit per antenna that was capped with GlcNAc (Fig. 2B; Glycan ID 557 and 573). Similar structures in the array (Supplementary Table 1) that lack the terminal GlcNAc cap on LacNAc were not efficient ligands for rBGL. This suggests that in the context of this class of N-glycan epitope, rBGL preferentially binds to the terminal GlcNAc that caps LacNAc or poly-LacNAc repeats and not GlcNAc internal to poly-LacNAc chains. Furthermore, linear polymers of β1,4-linked GlcNAc (Glycan IDs 189, 190, and 191) were also not bound by rBGL. Considered together, these observations support the conclusion that rBGL binds to β-GlcNAc in the terminal position of N-glycan antennae, likely with a preference for α-mannose, and to a lesser degree β-galactose, as the penultimate sugar.

**Table 1:** Human IgG *N*-glycans captured by EDGE UPLC-HILIC-FLR profiling with various BGLs.

Peak no.^a^	Glycan structure^b^	Retention time (min)	% Glycan binding^c^			
			rBGL	G49N	H71E	S48K
1		3.907	84	100	61	85
2		4.561	87	94	62	88
3		5.141	61	77	42	67
4		5.349	29	87	0	42
5		5.674	36	46	0	100
6		5.895	29	50	25	38
7		6.118	32	54	26	43
8		6.369	29	49	24	38
9		6.592	17	18	13	26
10		7.601	1	0	1	1
11		8.047	7	3	5	6
12		8.337	42	67	37	49
13		8.965	9	0	8	12
14		9.859	0	0	0	1
15		12.081	0	0	0	6
16		12.34	0	0	0	6

^a^A complex mixture of 2AB-labeled human IgG *N*-glycans was subjected to EDGE sample preparation and UPLC-HILIC-FLR profiling to visualize 16 chromatography peaks that are known to each contain single or multiple *N*-glycan structures.

^b^Sugar symbols are as shown in Fig. 2

^c^The percentage of each initial chromatography peak area that is recovered after lectin enrichment. It is calculated as described in “Materials and methods” section.

### rBGL substrate recognition

Crystal structures of the *Sclerotium rolfsii* lectin (SRL) in complex with monosaccharides have been previously reported^14^. SRL was separately crystallized in complex with GalNAc (PDB ID: 2OFD) and GlcNAc (PDB ID: 2OFE). SRL is predicted to have two separate monosaccharide binding sites, a “primary site” that associates with GalNAc and “secondary site” that associates with GlcNAc. rBGL and SRL share 64% sequence identity and a similar length (rBGL has a single-residue insertion compared to SRL). In addition, most residues that form both monosaccharide binding sites are strictly conserved with only Pro-102 of BGL being different from Asn-102 of SRL (Fig. 1B). Thus, SRL was used to generate structural models of rBGL-GalNAc and rBGL-GlcNAc binary complexes.

**Figure 3:**
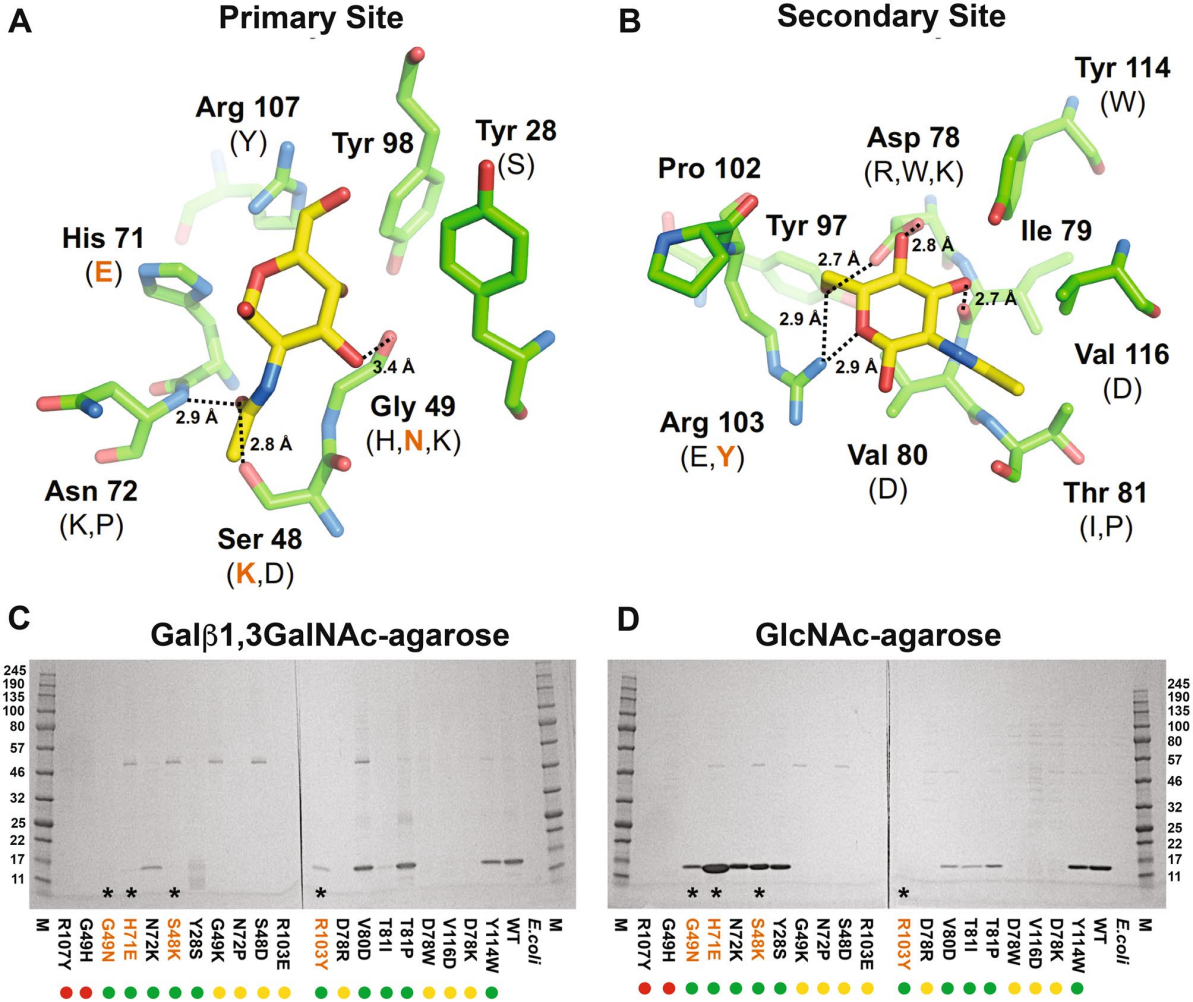
Modeled primary and secondary binding sites of rBGL. (**A**) Residues forming the primary binding site were mutated to specifically disrupt binding to GalNAc. (**B**) Secondary site residues were mutated to specifically disrupt binding to GlcNAc. Molecular graphic images were generated by V. Potapov using PyMOL^41^. All mutations tested are shown in parenthesis. Mutations that specifically abolish binding are highlighted in orange. Dotted lines indicate hydrogens bonds between the ligands and protein and the distance between donor and acceptor atoms is given in angstroms (Å). The overall structural model of rBGL is shown in Supplement Fig. S4. (**C**,**D**) Qualitative assessment of mutant lectin specificity. Computationally predicted mutants that disrupt each binding site were created by site directed mutagenesis and expressed in *E. coli*. Aliquots of each lysate were separately passed over Galβ1,3-GalNAc-agarose and GlcNAc-agarose columns. Proteins that bound to each column were eluted and separated by SDS-PAGE. Mutants G49N, H71E and S48K bound to GlcNAc but not Galβ1,3-GalNAc-agarose. Mutant R103Y bound to Galβ1,3-GalNAc but not GlcNAc-agarose. Recombinant BGL (WT) and *E. coli* extract were used as controls. Asterisks and colored text denote mutants with differential binding ability. To aid interpretation of binding trends, colored dots indicate the general level of expression of each mutant protein (green, soluble expression; yellow, poor/insoluble expression; red, no detected expression). M, broad range molecular weight protein standard (kDa, New England Biolabs catalog #P7712S). The uncropped gel images are shown in Supplement Fig. S11.

The rBGL-ligand structural models were used to investigate the biochemical function of each binding site. The binding pockets in rBGL for the primary site (GalNAc) and for the secondary site (GlcNAc) are shown in Fig. 3A and B, respectively. Candidate mutations were predicted to specifically disrupt the rBGL-ligand interaction at both the primary and secondary sites (see “Materials and methods”). The 10 resulting mutations for each binding site (Fig. 3A,B, Supplementary Table 2, Supplement Fig. S4) were chosen for characterization and qualitatively assessed for their ability to bind substrate-linked agarose resins (Galβ1,3GalNAc-α-agarose or GlcNAc-agarose) (Fig. 3C,D). Three mutations of the primary binding site (S48K, G49N, and H71E) each bound to GlcNAc-agarose but showed no binding to Galβ1,3GalNAc-α-agarose, suggesting that the function of the primary binding site had been disrupted. Conversely, one mutation of the secondary site (R103Y) yielded a protein that exclusively bound to Galβ1,3GalNAc-α-agarose, but not GlcNAc-agarose.

**Figure 4:**
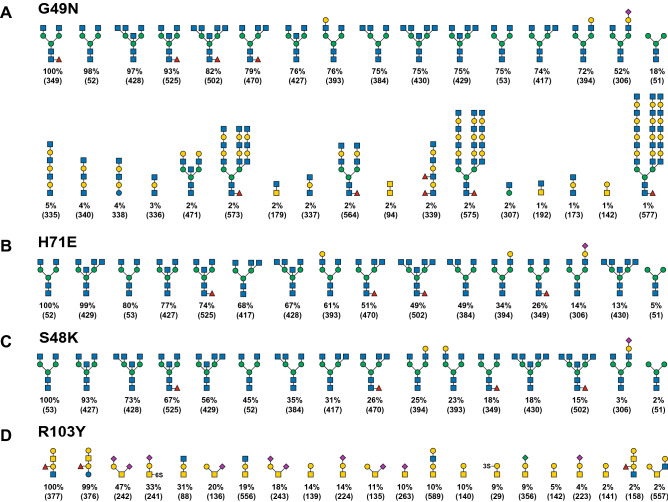
Oligosaccharide binding specificity of BGL mutant proteins using a mammalian glycan array. Fluorescently labeled proteins G49N, H71E, S48K, and R103Y were used to probe a mammalian glycan array at the Consortium for Functional Glycomics. (**A**–**D**) The specificity of each mutant was determined by testing its ability to bind to a printed array (version 5.3) consisting of 600 mammalian glycans. The structures of oligosaccharides bound by each protein are shown in decreasing order of ligand binding efficiency down to 1% binding relative to the strongest signal (see Supplementary Tables 3–6 for raw array data). Structures appearing more than once reflect identical glycans in the array that possess different chemical linkers. The CFG array’s Glycan ID numbers for each structure are shown in parentheses. Sugar symbols are as shown in Fig. 2.

The specificity of each of the four mutant rBGL proteins (S48K, G49N, H71E, and R103Y) was further assessed via binding to a printed array of 600 mammalian glycans (CFG version 5.3). Primary binding site mutants (S48K, G49N and H71E) robustly bound to all 15 array N-glycans bearing terminal GlcNAcβ1,2Man epitopes and a single paucimannose structure (Fig. 4A–C and Supplementary Tables 3–5). G49N also showed very weak association with some GlcNAc-terminated LacNAc-containing structures, whereas S48K and H71E did not. Similarly, the secondary binding site mutant (R103Y) exclusively bound short glycans bearing the Galβ1,3GalNAc epitope (Fig. 4D and Supplementary Table 6). Comparison of the top ten Galβ1,3GalNAc-containing ligands bound by wild-type rBGL or R103Y showed that eight glycans were recognized by both proteins (Figs. 2B and 4D). However, the relative binding efficiency of the R103Y mutant for some glycans (e.g., Neu5Acα2-3Galβ1-3(6S)GalNAcα-Sp8 and Galβ1-3GalNAcα1-3(Fucα1-2)Galβ1-4GlcNAc-Sp0) was different compared to wild-type rBGL, but was nearly unchanged for other ligands (e.g., Neu5Acα2-6(Neu5Acα2-3Galβ1-3)GalNAcα-Sp8). This may reflect a minor difference in ligand recognition by R103Y.

### Ligand binding site function

Surface plasmon resonance was used to further assess the function of the two ligand binding sites in rBGL and each of the four binding site mutants. Each of the rBGL proteins was attached to the surface of a separate sensor chip and tested for binding to GlcNAc or Galβ1,3-GalNAc (TF-antigen) using a twofold concentration series as described in the "Materials and methods". Response data was obtained for 8 replicate binding experiments for each protein with each ligand. The data was highly reproducible in each experiment and standard error was computed from the average of the replicate analyses.

**Figure 5:**
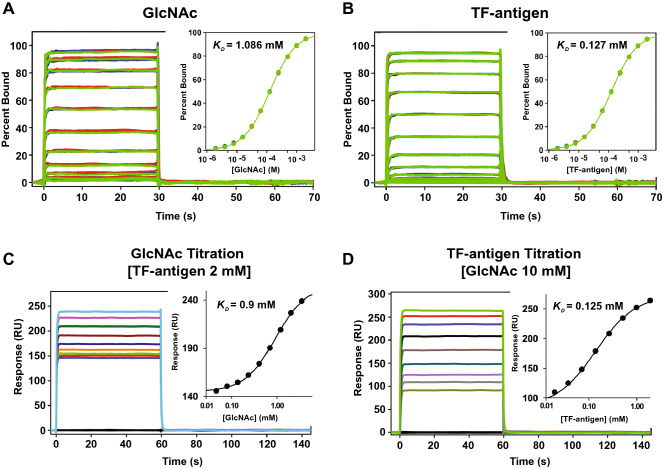
Ligand binding analysis of wild-type rBGL using SPR. (**A**) GlcNAc and (**B**) TF-antigen (Galβ1,3-GalNAc) were tested for binding to immobilized rBGL using a twofold concentration series up to 10 mM and 2 mM, respectively. Shown are sensorgrams captured for 8 replicate binding experiments (overlayed colored lines). Two competition binding experiments were run with the rBGL surface. (**C**) TF-antigen concentration was fixed at 2 mM while GlcNAc was tested in a twofold concentration series up to 5 mM. (**D**) GlcNAc concentration was fixed at 10 mM while TF-antigen was tested in a twofold concentration series up to 2 mM. For all experiments, equilibrium dissociation binding constants were determined by fitting data at equilibrium to a 1:1 interaction model using the software Scrubber 2. Fit curves are shown in insets.

For wild-type rBGL, the concentration series approached saturation for both GlcNAc and TF-antigen (Fig. 5A,B) and the equilibrium response data fit well to a 1:1 interaction model. GlcNAc bound to the rBGL surface with a *K*_*D*_ of 1.086 ± 0.008 mM, whereas TF-antigen bound with roughly ten-fold higher affinity (*K*_*D*_ of 0.127 ± 0.002 mM). Binding experiments were also conducted for each of the four binding site mutants. GlcNAc bound to primary binding site mutant surfaces S48K, G49N and H71E (*K*_*D*_ of 1.01 ± 0.02 mM, 0.91 ± 0.01 mM, 2.33 ± 0.08 mM, respectively) with similar strength as wild-type rBGL (Supplement Figs. S5A and S6A). No GlcNAc binding was observed for the secondary site mutant R103Y. In contrast, TF-antigen bound to the secondary site mutant R103Y surface (*K*_*D*_ of 0.105 ± 0.002 mM) with similar strength to rBGL (Supplement Figs. S5B and S6B). Very low affinity binding to the H71E surface (*K*_*D*_ of 17 ± 2 mM) was seen, and no significant binding to S48K and G49N surfaces was observed.

A ligand binding competition experiment was also performed for wild-type rBGL. In two parallel experiments, the rBGL surface was incubated with a fixed concentration of GlcNAc (10 mM) or TF-antigen (2 mM) to saturate one binding site. Binding of a second ligand was then tested in a twofold concentration series (up to 5 mM GlcNAc or 2 mM TF-antigen, respectively) (Fig. 5C,D). The rBGL surface bound to GlcNAc in the presence of TF-antigen with a *K*_*D*_ of 0.9 mM, essentially the same as GlcNAc alone (1.086 ± 0.008 mM). Similarly, TF-antigen bound the rBGL surface with a *K*_*D*_ of 0.125 mM, nearly identical to TF-antigen alone (0.127 ± 0.002 mM). These data show that each ligand selectively binds in the presence of the other, that both binding sites can be simultaneously occupied, and that there is no detectable allosteric communication between the two sites.

### Use of BGL mutants in N-glycan structural profiling

**Figure 6:**
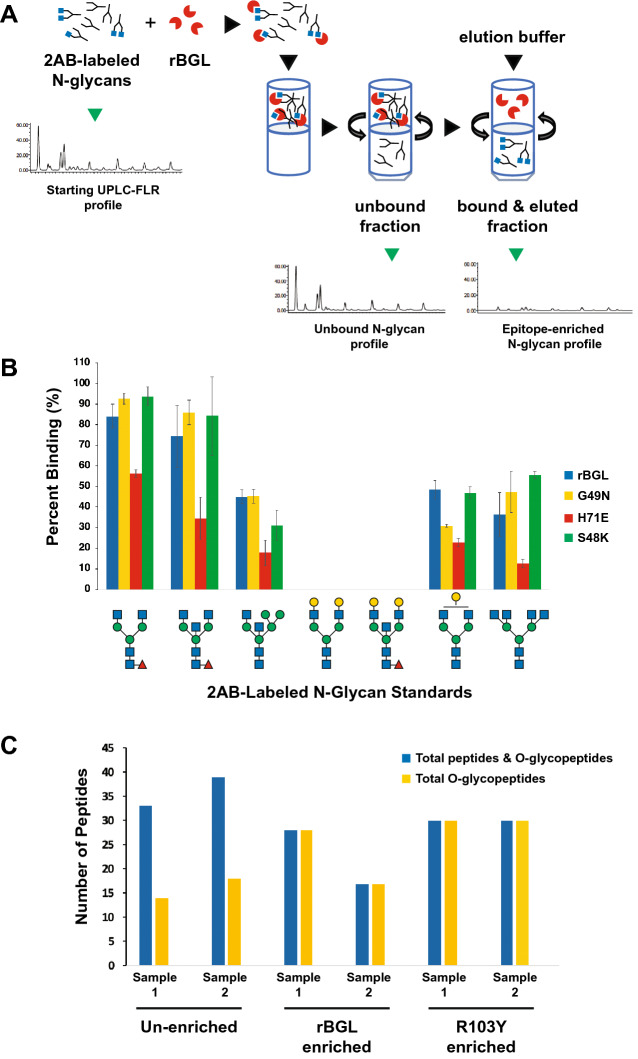
Use of rBGL and BGL mutants in analytical glycan enrichment schemes. (**A**) A schematic illustrating epitope-directed glycan enrichment (EDGE) sample preparation for UPLC-HILIC-FLR profiling^12^ (drawing by S. Vainauskas and C. Taron). A mixture of 2AB-labeled N*-*glycans is mixed with a highly specific lectin (e.g., rBGL). The mixture is applied to a centrifugal filter (MWCO 10–30 kDa) and spun to separate lectin-bound or unbound N*-*glycans. An elution buffer dissociates the lectin–glycan complex and N*-*glycans possessing the desired epitope (e.g., terminal GlcNAc) are recovered by centrifugation then subjected to UPLC-HILIC-FLR analysis. (**B**) EDGE UPLC-HILIC-FLR profiling of several 2AB-labeled N-glycan standards. Percent glycan binding (calculated as described in “Materials and methods”) is shown for rBGL and mutants G49N, H71E, and S48K. (**C**) Enrichment of O-glycosylated peptides/peptiforms from Pronase digested bovine fetuin before and after enrichment with rBGL or R103Y. Sample 1 and 2 represent replicate samples that were each separately digested with Pronase and subjected to lectin enrichment. Blue bars represent the total number of peptides identified (unglycosylated peptides and O-glycopeptides). Yellow bars represent the number of unique O-glycopeptides/peptiforms identified in each sample (see Supplementary Table 7 for the O-glycan structures observed).

The ability of wild-type rBGL and the mutants S48K, G49N and H71E to bind to terminal β-GlcNAc on N-glycans has potential application in N-glycan structural profiling. A common approach for N-glycan analysis involves their enzymatic release from a glycoprotein (often a biologic drug or serum IgG) using the enzyme PNGase F, fluorescent labeling of the liberated N-glycans, and their separation using HILIC-based ultra-performance liquid chromatography with inline fluorescence detection (UPLC-HILIC-FLR). In complex samples where numerous N*-*glycan species are present, individual chromatography peaks may be comprised of multiple glycan structures. Some highly specific lectins can be used to reduce sample complexity by permitting subtraction of structures that bear a specific sugar epitope using a filter-assisted sample preparation technique (Fig. 6A). We defined this method for UPLC-HILIC-FLR analyses^12^ and have given it the acronym epitope-directed glycan enrichment (EDGE) UPLC-HILIC-FLR profiling. It is noteworthy that not all lectins perform well in EDGE sample preparation, and individual lectins must be empirically tested for their suitability in this application.

We compared the performance of wild-type rBGL and the mutants S48K, G49N and H71E in EDGE UPLC-HILIC-FLR profiling. In a first experiment, each lectin was assessed for its ability to quantitatively enrich fluorescently labeled N-glycan standards containing different numbers of terminal β-GlcNAc residues as described in Materials and methods. The lectins all recognized N-glycans having one or more terminal β-GlcNAc residues. Mutants G49N and S48K also gave similar levels of enrichment as wild-type rBGL, while H71E showed weaker glycan recovery (Fig. 6B). A second similar experiment measured the ability of each lectin to quantitatively recover GlcNAc-terminated N-glycans from a complex glycan mixture derived from human IgG (Table 1). rBGL and mutants G49N and S48K performed nearly identically, although some weak binding of glycans with no terminal GlcNAc residues (peaks 14–16) was seen for S48K. We conclude that G49N is best-suited for use as a highly specific binding reagent for EDGE UPLC-HILIC-FLR N-glycan structural profiling.

### Use of rBGL in O-glycopeptide sample enrichment

The ability of wild-type rBGL to bind to a variety of mucin-type O-glycans regardless of their degree of structural complexity suggests it could be used as an enrichment reagent in proteomics analyses of O-linked glycopeptides. In a typical bottom up proteomics method, a protein or mixture of proteins is digested with a protease, and glycopeptides are subjected to chromatographic separation and analysis by mass spectrometry. Here we utilized the model O-glycoprotein bovine fetuin and digested it with Pronase to generate a peptide/glycopeptide mixture. This mixture was subjected to O-glycopeptide enrichment using either wild-type rBGL or the R103Y mutant as described in Materials and methods. An unenriched sample was run as a control, and all enrichments were performed on two biological replicate samples. All mixtures were analyzed using high-resolution LC–MS/MS on an Orbitrap Q-Exactive with HCD fragmentation. The obtained tandem mass spectra were searched using Byonic software to identify the number and composition of unique unglycosylated peptides and O-glycopeptides (peptides/peptiforms possessing unique O-glycan structures) in each sample (Fig. 6C).

In both wild-type rBGL and R103Y enriched samples, 100% of the observed peptides/peptiforms were O-glycosylated compared to 42–46% in the unenriched samples (Fig. 6C), indicating that lectin binding was both efficient and highly selective for O-glycopeptides. To determine if O-glycan structural composition biased the enrichment, O-glycan structures were predicted from the collected mass data. The major species observed in all samples were mono- and di-sialylated Core 1 and Core 2 O-glycans (Supplementary Table 7). Additionally, less abundant TF-antigen structures containing N-glycolylneuraminic acid (Neu5Gc) were observed in all non-enriched and enriched samples. These data support the conclusion that both wild-type rBGL and R103Y broadly recognize the repertoire of O-glycan structures present on bovine fetuin peptides. Furthermore, this experiment suggests that rBGL enrichments may be advantageous for analysis of O-glycopeptides from more complex samples.

## Discussion

The lectin BLL from the mushroom *Boletopsis leucomelaena* was previously shown to selectively bind N-glycans bearing terminal GlcNAc residues^9–11^. However, it’s protein sequence has not been previously reported. To enable its recombinant production, we identified a transcript encoding a BLL functional ortholog (termed BGL) from a closely related *Boletopsis* species (*B. grisea*). The BGL peptide sequence showed strong homology to mushroom fruit body lectins from the protein family PF07367. Interestingly, members of this family bind Tn-antigen (GalNAc-α-Ser) or TF-antigen (Galβ1,3GalNAc) epitopes present in mucin-type O-glycans^14,15,18,20^ but less is known about their ability to bind terminal GlcNAc on N-glycans. Additionally, these proteins possess two ligand binding sites per protein monomer^14,15,20^. Thus, in this study, we used recombinant BGL as a model to explore the function and specificity of each ligand binding site, and to construct highly specific lectin mutants for application in glycoanalytical workflows.

Creation of a structural model for wild-type rBGL permitted construction of mutant rBGL proteins defective in either the primary or secondary ligand binding sites. Mammalian glycan microarray analysis was used to compare the oligosaccharide binding specificities of wild-type rBGL and each of the mutants. Wild-type rBGL showed a clear ability to bind oligosaccharides containing either of two different epitopes; Galβ1,3-GalNAc found in mucin-type O-glycans and terminal GlcNAc in N-glycans (preferably in the context of GlcNAcβMan). Additionally, binding analysis with each of the single binding site rBGL mutants highlighted that the two binding sites have distinct oligosaccharide binding preferences with the primary site recognizing Galβ1,3GalNAc and the secondary site recognizing primarily GlcNAcβMan.

The oligosaccharide binding specificity of wild-type rBGL appeared different from that previously reported for XCL^21^ and SRL^22^. All three proteins share the ability to bind the TF-antigen (Galβ1,3GalNAc) epitope of O-glycans ^14,23^. However, in contrast to rBGL, XCL and SRL were not previously described as efficient binders of N-glycan terminal GlcNAc. This was somewhat surprising as the XCL and SRL proteins share 57 and 64% sequence identity with rBGL, respectively. Additionally, SRL’s residues that comprise its primary and secondary ligand binding site constellations are highly conserved within rBGL and were the basis for our structural model. Thus, we re-examined XCL and SRL glycan microarray binding data obtained from the Consortium for Functional Glycomics (CFG) data archive. SRL was first tested using CFG array v2.1 in 2006 (cfg_rRequest_637). This early array contained only a single N-glycan bearing terminal GlcNAcβMan (ID #51). However, it was among the top five substrates bound by SRL in that experiment. Later CFG binding experiments with SRL and XCL using array v4.1 (cfg_rRequest_2025) and v5.0 (cfg_rRequest_2617) showed efficient binding to 13 and 16 N-glycans having terminal GlcNAcβMan, respectively. Thus, as the N-glycan content of the CFG array improved over time, the terminal GlcNAc specificity of these lectins has become more apparent, and is consistent with our current observations with wild-type rBGL.

Surface plasmon resonance gave further insight into the function of rBGL’s two ligand binding sites. SPR with wild-type rBGL showed that both TF-antigen (*K*_*D*_ = 0.127 ± 0.002 mM) and GlcNAc (*K*_*D*_ = 1.086 ± 0.008 mM) were efficiently bound with an ~ tenfold preference for TF-antigen binding. The same analysis with each binding site mutant showed that rBGL’s primary and secondary sites were highly selective for binding TF-antigen and GlcNAc, respectively. SPR titration binding experiments with wild-type rBGL, where one ligand was assessed for binding in the presence of a saturating concentration of the second ligand, showed that each ligand efficiently bound in the presence of the other and that both sites could be simultaneously occupied (at least with small ligands). Additionally, the binding equilibrium of rBGL with each ligand in the presence of the other was nearly identical to that of each ligand alone indicating there is no detectable allosteric interaction between the two sites.

Some studies have indicated that fruit body lectins from PF07367 are involved in mushroom defense against predation and have insecticidal properties^21,24–27^. While the precise molecular mechanism of their entomotoxicity is not entirely clear, the process generally involves a lectin binding to glycans on the surface of insect gut epithelial cells and inducing apoptosis^25^. Our specificity analysis of rBGL shows that it binds to core epitopes abundantly found in insect glycans. For example, known insect O-glycans possess a TF-antigen core^28^, whereas, typical insect neutral N-glycans lack outer arm galactose and sialic acid, and possess terminal GlcNAcβ1,2Man^29,30^. The bivalent nature of these lectins further suggests a role in crosslinking surface glycoproteins, possibly as part of an insecticidal mechanism. Such a concept has precedent in mammals where multivalent lectins (galectins) bind cell surface β-galactoside-containing glycans and modulate a variety of important cellular functions such as cell proliferation, cell adhesion, tumor progression, and apoptosis^31,32^. Galectins crosslink cell surface glycoproteins into raft-like membrane domains (lattices) that hold certain glycoproteins (e.g., signaling receptors) in close proximity while excluding others. Lattice crosslinking is made possible by the ligand binding multivalency of secreted galectins, that typically possess two or more carbohydrate recognition sites. It remains to be determined if both ligand binding sites and crosslinking are similarly important for the biological function of PF07367 proteins, however, the monospecific rBGL mutants produced in this study could be used to further investigate this notion in an insect cell model.

In summary, our study assigns BLL/BGL to the mushoom fruit body protein family (PF07367). We show that BGL has two independently functioning ligand binding sites that possess markedly different specificities. Its primary binding site selectively binds a core motif of mucin-type O-glycans (Galβ1,3GalNAc), while its secondary binding site preferentially recognizes the complex N-glycan epitope GlcNAcβMan. Our study further extends the understanding of glycan binding and specificity for PF07367 proteins and hints at a possible crosslinking role they may perform in mushroom defense. Finally, we show that single binding site mutant rBGL proteins have promise as novel highly specific tools for use in glycoanalytical workflows.

## Materials and methods

### Materials

Chemical reagents, solvents and monosaccharides were from MilliporeSigma (St. Louis, MO). 2AB-labeled oligosaccharide standards (NGA2F, NGA2FB, M5A1B, NA2, NA2F, NA2G1F, NGA4) were from Agilent Technologies, Inc. (Santa Clara, CA). Galβ1,3GalNAc-α- (TF-antigen) and GalNAc-α-Ser (Tn antigen) were from Carbosynth (Compton, UK). The carbohydrates GlcNAc and TF-antigen were immobilized to a 6% cross-linked divinyl sulfone activated agarose bead. Ligand density was not determined.

The putative Kurokawa mushroom was obtained from Wilson Farms (Lexington, MA). Proteinase K was from New England Biolabs (Ipswich, MA). Human serum IgG antibody was obtained from MilliporeSigma. Phosphate-buffered saline (PBS, pH 7.4) was from Teknova (Hollister, CA). DNA primers used in this study are shown in Supplementary Table 8.

### Mushroom speciation

To obtain mushroom spore prints, cross sections of fruiting bodies were cut, and the slices placed with spore tubes facing down on a glass microscope slide at ambient temperature for at least 14 h. The mushroom slices were removed from each slide, a drop of deionized water was added to each print and covered with a glass coverslip. Microscopy was performed on a Zeiss Axiovert 200 M microscope (Zeiss, Germany) at 400× magnification.

Molecular speciation of the sourced mushroom was performed by sequencing of the fungal internal transcribed spacer (ITS) rRNA region as described^13^. Genomic DNA was isolated from 100 mg of mushroom fruiting body using the OmniPrep kit (G-Biosciences, St. Louis, MO). PCR amplification of the ITS locus from genomic DNA was performed using the fungal-specific primer ITS1-F^33^ and universal primer ITS4^34^.

The ~ 700 bp amplicon containing the fungal ITS regions flanking 5.8S rRNA was subjected to Sanger nucleotide sequencing using the ITS1-F and ITS4 primers. The obtained nucleotide sequence was used to query GenBank sequences using the BLASTN algorithm at the National Center for Biological Information website (http://blast.ncbi.nlm.nih.gov/Blast.cgi) and search results were filtered for fungal sequences.

### *Boletopsis grisea* transcript sequencing and lectin cDNA identification

Approximately 100 mg of mushroom fruit body were frozen with liquid nitrogen and homogenized by grinding. Total RNA was purified from the powder using the RNeasy Plant Mini Kit (Qiagen, Chatworth, CA). An mRNA transcript library suitable for Illumina sequencing was created using the NEBNext Ultra Directional RNA Library Prep Kit for Illumina (New England Biolabs, Ipswich, MA). Paired-end deep sequencing (2× 100 bp) was performed on a MiSeq sequencer (Illumina, San Diego, CA). Adapter sequences were first removed from the raw reads using *CutAdapt*^35^. Trimmed reads were assembled using Trinity software^17^. Assembled transcript sequences were deposited in the NCBI Transcriptome Shotgun Assembly Sequence Database under accession number GEZR00000000. Raw reads were deposited in the NCBI Sequence Read Archive under accession number SRR090126.

The program BLASTX was used to translate the *B. grisea* transcripts in all six reading frames and compare the deduced amino acid sequences to a previously reported internal peptide sequence (GGSGTSGTIR) derived from the *B. leucomelaena* (BLL) lectin^9^. A single transcript was identified (Genbank No. KT315924).

### Assembly of an *E. coli* BGL expression vector

For construction of a recombinant expression plasmid of BGL in *E. coli*, its ORF sequence (GenBank KT315924) was inserted into pET21a(+) via the Gibson Assembly Cloning Kit (New England Biolabs). The 432 bp BGL coding sequence was PCR-amplified from *B. grisea* cDNA using primers BGL-F and BGL-R (Supplementary Table 8). The NEBuilder Assembly Tool (http://nebuilder.neb.com/) was used for design of Gibson Assembly primers.

### Expression and purification of recombinant BGL

Luria–Bertani (LB) medium supplemented with 100 µg/mL ampicillin was inoculated with NEB T7 Express *E. coli* cells carrying the pET21a-BGL plasmid and grown at 37 °C until the OD_600_ reached 0.4. Isopropyl β-D-thiogalactoside (IPTG) was added at this point to induce expression and the culture was incubated at 16 °C overnight with shaking. Cells were harvested by centrifugation, re-suspended in column buffer (50 mM sodium phosphate, 300 mM NaCl and 1 mM EDTA, pH 8.0), and lysed by passing through a TS Series cell disruptor (Pressure Biosciences, Inc., South Easton, MA.). The lysate was loaded onto a GlcNAc-agarose column that had been pre-equilibrated with column buffer. The column was washed with wash buffer (50 mM sodium phosphate, 500 mM NaCl and 1 mM EDTA, pH 8.0) and bound protein was eluted with column buffer containing 0.25 M GlcNAc. Fractions containing purified recombinant BGL were identified by SDS-PAGE and stained with SimplyBlue SafeStain (Thermo-Fisher Scientific). Purified BGL was quantified by measuring optical density at 280 nm using a NanoDrop 2000 spectrophotometer (Thermo-Fisher Scientific). The percent solution extinction coefficient (e1%) of 17.10 (g/100 mL)^−1^ cm^−1^ was calculated using the ExPASy ProtParam tool (https://web.expasy.org/protparam/).

### Designing structure-guided ligand binding mutations

Structural models of BGL-GalNAc and BGL-GlcNAc complexes were built based on the crystal structures of *Sclerotium rolfsii* lectin, SRL in complex with GalNAc (PDB ID: 2OFD; ligand NGA 144) and GlcNAc (PDB ID: 2OFE; ligand NAG 145) using the Homology Modeling workflow in the Schrodinger molecular modeling software with default settings^36^. The ligand molecules were retained in the same position and conformation as observed in the template crystal structures and included during model building. The binding pockets for GalNAc and GlcNAc were defined as any protein residue within 4.5 Å from the ligand. For each ligand, each residue in the corresponding binding pocket was mutated to all other possible residues and the effect of mutation on ligand binding was evaluated using docking simulations. The simulations were performed with Induced Fit Docking using the standard protocol with default settings^37^. The receptor grids were centered on the ligand molecule as defined in the structural models. During initial Glide docking, the side chain of mutated binding pocket residue was removed. The effect of mutation on binding was estimated based on Prime MM-GBSA ΔG_binding_ values, which were computed as part of the simulations by comparing binding energy of resulting single-residue mutant complexes to the binding energy of the complex with the native residue at the respective position. The lowest scoring pose for each complex was used in comparison. For each binding pocket, mutations were ranked by their destabilizing effect on binding and filtered to avoid more than 3 mutation candidates at any given position. Any mutations predicted to have a destabilizing effect on the protein itself were also excluded. After filtering, 10 mutations in each pocket were selected for experimental testing (Supplementary Table 2). For the secondary binding site (GlcNAc), one of the chosen mutations (Y114W) was included as a control to test the prediction method as it was predicted to be the most stabilizing for ligand binding.

### Site directed mutagenesis of rBGL

Site-directed BGL mutants were generated using the Q5 Site-Directed Mutagenesis Kit (New England Biolabs) as recommended by the manufacturer. Primers were designed using NEBaseChanger (nebasechanger.neb.com) (Supplementary Table 8). The pET21a-BGL vector was used as template for PCR amplifications. Kinase, ligase and DpnI (KLD) treatment was performed on the PCR products as described in the kit. Mutant plasmids were introduced into NEB 5-alpha *E. coli* cells (New England Biolabs) and isolated plasmids were sequenced to verify the incorporation of desired mutations.

### Mutant rBGL affinity chromatography assays and purification

To assess the binding specificity of BGL mutants, a 10 mL culture of NEB T7 Express cells carrying each pET21a-mutantBGL plasmid was grown and induced as described above. Cells were harvested by centrifugation, re-suspended in 1 mL of column buffer (50 mM sodium phosphate, 300 mM NaCl and 1 mM EDTA, pH 8.0), and sonicated (Qsonica, Newtown, CT). Lysed cells were centrifuged and equal amounts of each lysate (as measured on a NanoDrop 2000 spectrophotometer) was separately passed over a 50 µL TF-antigen agarose column and a 125 µL GlcNAc agarose column, both of which had been pre-equilibrated with 1 mL of column buffer. Bound protein was eluted with 20 mM NaOH (TF-antigen column) or 0.25 M GlcNAc (GlcNAc column). The eluted fractions were analyzed by separation on an SDS-PAGE to qualitatively assess each mutant’s ability to bind each resin.

To purify larger amounts of mutants H71E, G49N, S48K and R103Y, large scale LB-amp cultures (4–6 L) of each mutant were grown and induced as described for expression of rBGL above, and purified to > 90% purity. Mutants H71E, G49N and S48K were purified by passage over GlcNAc-agarose columns while R103Y was purified using a TF-antigen agarose column (Supplement Fig. S7). Pooled elution fractions were dialyzed in 1× PBS, pH 7.4. Protein concentrations were determined by Bradford assay (Supplement Fig. S8) and protein purity of rBGL and mutants was also assessed by Western blot analysis (Supplement Fig. S9).

### Surface plasmon resonance analysis of glycan binding

Surface plasmon resonance (SPR) was used to assess the binding strength of recombinant BGL and the BGL mutants to GlcNAc and Galβ1,3GalNAc antigen (TF-antigen) using a Biacore 4000 biosensor (GE Healthcare Life Sciences). BGL proteins were coupled to a CM7 sensor chip (10,000–20,000 RU) at 40 µg/mL in 10 mM NaAc, pH 4.5 using standard NHS/EDC activation. Running buffer contained PBS at pH 7.4 and data were collected at 25 °C. GlcNAc and TF-antigen were tested for binding using a twofold concentration series up to 10 mM and 2 mM, respectively. Samples were injected at the instruments maximum flow rate (30 µL/min). Response data were collected for 8 replicate studies. Data were processed by subtracting responses from a reference surface without BGL coupled (Scrubber 2, Biologic Software Pty Ltd) and normalized for amount bound and R_max_. Report points taken at equilibrium were fit to a simple 1:1 interaction model to determine the equilibrium dissociation binding constants. A competition study was run with GlcNAc fixed at 10 mM while TF-antigen was tested in a twofold concentration series up to 2 mM. An additional competition study was run with TF-antigen fixed at 2 mM while GlcNAc was tested in a twofold concentration series up to 5 mM.

### Mammalian glycan microarray analysis

Purified recombinant BGL and four mutants were labeled with DyLight 488 NHS Ester (Thermo-Fisher Scientific) according to the manufacturer's instructions and dialyzed against 20 mM Tris–HCl, pH 7.4, 150 mM NaCl using 3000 MWCO dialysis membranes. DyLight 488 labeled protein was used for glycan array screening at the Consortium for Functional Glycomics Protein–Glycan Interaction Core (Harvard Medical School, Boston, MA).

The specificity of BGL and 4 mutants was determined by screening each protein’s binding to the printed array (versions 5.2 and 5.3 consisting of 609 and 600 mammalian glycans, respectively). Detailed information about the structures and linkers of these glycans can be found at https://glycopattern.emory.edu^23^. The printed array was probed with 200 μg/mL of each lectin diluted in 20 mM Tris–HCl, pH 7.4, 150 mM NaCl, 2 mM CaCl_2_, 2 mM MgCl_2_, 0.05% Tween 20, 1% BSA, in 6 replicate binding experiments. The highest and lowest point from each set of six replicates was discarded, and the average RFU value of 4 replicates, the standard deviation, and % CV (% CV = 100 × Standard Deviation/Mean) were calculated.

### Epitope directed glycan enrichment UPLC profiling

Substrates for EDGE profiling experiments were either individual N-glycan standards (Prozyme) or N-glycans released from polyclonal human IgG (MilliporeSigma). To release N-glycans from human IgG, a 50 uL reaction containing IgG (120 µg) and 1 µL of Rapid PNGase F (New England Biolabs) in the supplied reaction buffer was incubated for 10 min at 50 °C. Released IgG N-glycans and N-glycan standards were fluorescently labeled with 2-aminobenzamide (2AB) as previously described^12^. Lectin capture of labeled N-glycans was performed as previously described^12^ with slight modifications. Briefly, 2AB-labeled N-glycan standards (4 pmol) or 2AB-labeled human IgG glycans (~ 108 pmol) were incubated with 120 μg of BGL in 120  μL of 10 mM Tris–HCl, pH 8.0 for 3 h at RT. Reaction mixes were transferred to Microcon-30 (Ultracel YM-30, MilliporeSigma) centrifugal concentration devices that had been washed with 500 μL of deionized water. The tubes were centrifuged for 5 min at 11,000×*g*, and the filtrates collected. The filters were washed twice with 120 μL of 10 mM Tris–HCl, pH 8.0 buffer, centrifuged as above, and the washes combined with the initial filtrate. Pooled fractions contain N-glycans that did not bind to wild-type rBGL and flowed through the device. To elute rBGL-bound N-glycans, the filter devices were transferred to new collection tubes and 120 μL proteinase K mix (15 units of proteinase K without glycerol in 10 mM Tris–HCl, pH 8.0; New England Biolabs) was added to each device. The reaction was incubated at 37 °C overnight. The concentrators were again centrifuged for 5 min, washed twice, and filtrates pooled to recover N-glycans that had been bound to BGL. Pooled fractions were dried using vacuum evaporation and each sample was dissolved in 6 μL of deionized water. For UPLC-HILIC-FLR analysis, each sample was mixed with 14 μL acetonitrile (the final ratio of acetonitrile:water was 7:3).

2AB-labeled N-glycans were separated by UPLC using a Waters Acquity BEH glycan amide column (2.1 × 150 mm, 1.7 μm) on a Waters H-Class ACQUITY instrument (Waters Corporation) equipped with a quaternary solvent manager and a fluorescence detector. Solvent A was 50 mM ammonium formate buffer pH 4.4 and solvent B was acetonitrile. The gradient used was 0–1.47 min, 30% solvent A; 1.47–24.81 min, 30–47% solvent A; 25.5–26.25 min, 70% solvent A; 26.55–32 min, 30% solvent A. The flow rate was 0.561 mL/min. The injection volume was 18 μL and the sample was prepared in 70% (v/v) acetonitrile. Samples were kept at 5 °C prior to injection and the separation temperature was 40 °C. Fluorescence was measured with excitation and emission wavelengths of 330 nm and 420 nm, respectively. The data collection rate was 20 Hz. All data was processed using Waters Empower 3 chromatography workstation software. In EDGE UPLC-HILIC-FLR profiling experiments, percent binding was computed by dividing the relative area of a desired peak in the bound/eluted fraction by the peak’s total area in both bound/eluted and unbound fractions.

### Generation and enrichment of O-glycopeptides

Fetuin (200 μg, MilliporeSigma) was heated for 15 min at 98 °C in 250 μL denaturing buffer (0.1% SDS, 40 mM DTT in 20 mM Tris–HCl, pH 8.0). The sample was transferred to a Nanosep 10 K centrifugal devices and spun for 5 min at 11,000×*g*. The filtrate was discarded, and the filter was washed twice with 500 μL of 20 mM Tris–HCl, pH 8.0. After washing, 120 μL of protease solution [20 mM Tris–HCl, pH 8.0 containing 10 mM CaCl_2_ and 0.5 mg/mL Pronase (Roche Diagnostics, Indianapolis, IN)] was added to the filter and incubated for 24 h at 42 °C. Glycoprotein hydrolysate was collected by centrifugation (5 min at 11,000×*g*) and dried by vacuum evaporation. The dried glycoprotein hydrolysate was dissolved in 200 μL 20 mM Tris–HCl, pH 8.0 (1 μg/μL).

Fetuin hydrolysate (25 μg) was mixed with 50 μg of rBGL lectin (wild-type or R103Y mutant) in 20 mM Tris–HCl, pH 8.0 in a total reaction volume of 150 μL, and incubated for 1 h at RT. The reaction mix was transferred to a Nanosep 10 K filter device and centrifuged for 5 min at 11,000×*g*. The filtrate was discarded, and the filter was washed twice with 400 μL of 20 mM Tris–HCl, pH 8.0. Then, 120 μL elution buffer (0.2% formic acid, 50% acetonitrile) was added to the filter and incubated for 30 min at RT. The eluate was collected by centrifugation (5 min at 11,000×*g*) and dried by vacuum evaporation.

### Analysis of enriched O-glycopeptides

Peptides were dissolved in water to make 0.5 μg/μL (or equivalent of protein hydrolysate for enriched fraction). Samples were directly injected on a reversed phase 1.6 μm C18 fused silica column (I.D. 75 μm, O.D. 360 μm × 25 cm length) packed into an emitter tip (IonOpticks, Australia). The column was placed into a column oven integrated into the mass spectrometer ion source (Sonation Column Oven). The flow rate for analysis was 400 nL/min with buffer A (water, 0.1% formic acid) and buffer B (acetonitrile, 0.1% formic acid). The gradient conditions were: 2 min at 2% B, 30 min to 40% B, 5 min to 70% B, 2 min at 70% B with 1 min return to 2% B and a total of 40 min analysis time. The temperature was set at 40 °C. The Easy-nLC 1000 was coupled with a QExactive Hybrid Quadrupole-Orbitrap mass spectrometer. The most abundant 10 ions were fragmented in the stepped normalized collision energy: NCE 20, NCE 30 and NCE 40.

Glycopeptide MS/MS fragment spectra were searched against selected protein and glycan databases using Byonic software v3.4-55 × 64 (Protein Metrics Inc.)^38^. Searches were performed using precursor ion mass tolerance of 10 ppm, fragment ion mass tolerance of 0.02 Da, non-specific cleavage at C-term with any number of missed cleavages. Variable modifications used in searches: oxidation of Met (+ 15.994 Da), deamidation at Asn, Gln (+ 0.984 Da) and with the defined O-glycan database (13 glycans). Searches were performed against defined glycoproteins to narrow the search space. The searches were filtered using strict criteria (PEP 1D < 0.05; Delta Mod Score > 40). All glycopeptides were further manually validated using oxonium ions *m/z* 204.087 (HexNAc), 274.092/292.103 (Neu5Ac), and Neu5Gc-containing glycopeptides were validated by searching for the diagnostic ions *m/z* 290.090/308.090.

## Supplementary Information

Supplementary Information 1.

Supplementary Information 2.

## Abbreviations

BLL: *Boletopsis leucomelaena* lectin
BGL: *Boletopsis grisea* lectin
rBGL: Recombinant wild-type BGL
SRL: *Sclerotium rolfsii* lectin
XCL: *Xerocomus chrysenteron* lectin
GlcNAc: *N-*Acetylglucosamine
GalNAc: *N-*Acetylgalactosamine
Gal: d-Galactose
Man: d-Mannose
TF-antigen: Galβ1,3GalNAc-α-
Tn-antigen: GalNAc-α-
ITS: Internal transcribed spacer
RFU: Relative fluorescence units
EDGE: Epitope directed glycan enrichment
IPTG: Isopropyl β-d-thiogalactoside
SPR: Surface plasmon resonance
