# Supplementary material for: A bi-specific lectin from the mushroom Boletopsis grisea and its application in glycoanalytical workflows
Source: Sci Rep. 2021 Jan 8;11:160. doi: 10.1038/s41598-020-80488-7 (PMC7794217; doi:10.1038/s41598-020-80488-7)
Supplement: Supplementary file 1 — Supplementary Information 1. [file 41598_2020_80488_MOESM1_ESM.pdf]

Supplemental Information

A bi-specific lectin from the mushroom *Boletopsis grisea* and its application in glycoanalytical workflows

Mehul B. Ganatra, Vladimir Potapov, Saulius Vainauskas, Anthony Z. Francis, Colleen M. McClung, Cristian I. Ruse, Jennifer L. Ong and Christopher H. Taron*

New England Biolabs, Inc., 240 County Road, Ipswich, MA 01938, USA

*Corresponding Author: Christopher Taron, 240 County Road, Ipswich, MA 01938, USA, Phone: (978) 380-7207, Fax: (978) 921-1350, Email: taron@neb.com

This document provides information supplemental to the main text, in the following sections:

A - Supporting Experimental Section

B - Supporting Tables and Figures

A - Supporting Experimental Section

Native lectin purification

Purification of the native mushroom GlcNAc-binding lectin was conducted using a procedure described by Koyama *et al*. with some modifications. Fruit body (175 g) was homogenized in 200 mL of Buffer A (50 mM Tris-HCl, pH 7.5 containing 0.1 M NaCl, 1 mM EDTA, and Complete EDTA-free Protease Inhibitor Cocktail (Roche Diagnostics, Indianapolis, IN) at a concentration of 1 tablet per 50 mL buffer) using an electric tissue grinder. The homogenate was centrifuged for 10 min at 13,000 x *g*. Ammonium sulfate was added to the supernatant to 70% saturation. Precipitated proteins were dissolved in 30 mL Buffer A and residual insoluble material was removed by centrifugation for 10 min at 13,000 x *g*. The protein suspension was dialyzed using a 6-8 kDa MWCO membrane (Thermo-Fisher Scientific, Chelmsford, MA) against 3 L Buffer A lacking protease inhibitors for 2 days (with the buffer being changed after 24 h). Following dialysis, the sample was passed through a 0.22 μm filter. A 10 mL GlcNAc-agarose column was equilibrated with 100 mL Buffer A after which the dialyzed sample was applied. The column was washed with 70 mL Buffer B (50mM Tris-Cl, pH 7.5 containing 0.3 M NaCl, and 1 mM EDTA). The GlcNAc-binding lectin was eluted from the column in Buffer A containing 0.25 M GlcNAc in 4 mL fractions. Fractions containing the purified protein were analyzed on a 10-20% Tris-glycine SDS-PAGE gel (Invitrogen, Carlsbad, CA) using SimplyBlue SafeStain (Thermo-Fisher Scientific). The BGL containing fractions were pooled and dialyzed against 10 mM Tris-HCl, pH 7.5 using a Slide-A-Lyzer Dialysis Cassette (3.5K MWCO) (Thermo-Fisher Scientific).

Reference:
Koyama, Y. *et al.* Apoptosis induction by lectin isolated from the mushroom *Boletopsis leucomelas* in U937 cells. *Biosci. Biotechnol. Biochem.* **66**, 784-789 (2002).

Liquid chromatography mass spectrometry analysis (LC-MS)

The lectin samples were diluted to 100 ng/μl in 0.1% formic acid and analyzed by reverse phase liquid chromatography and electrospray ionization time-of-flight (ESI-TOF) mass spectrometry. A reverse phase HPLC-Chip (Agilent Technologies) was used for the separation of proteins (Vollmer & Goor, 2009). The custom chip contained an integrated trapping column with a 40 nl capacity, a separation column (75 μm x 150 mm), both packed with PLRP-S 5 μm particles (1000 Å pore size), and a nano-ESI emitter. The proteins were loaded onto the chip trapping column at 4 μl/min and eluted over the separation column at a flow rate of 500 nl/min using an Agilent 1200 series nano LC coupled to an Agilent ChipCube and 6210 series ESI-TOF mass spectrometer. The column was equilibrated with 15% acetonitrile/0.1% formic acid in water. For each protein, one μL of sample was injected onto the column. After two minutes, the protein was eluted over a ten-minute linear gradient from 15% to 90% acetonitrile (held at 90% acetonitrile

for five minutes). The proteins eluted at approximately thirteen minutes after injection. The mass spectra were acquired from 100 to 3200 m/z using an ionization energy of 1800 V, fragmentation energy of 215 V and drying gas of 325°C at 4.0 L/min. The spectra were extracted and deconvoluted with Agilent MassHunter Qualitative Analysis using BioConfirm B 2.0.2 software with a mass range of 10k to 50k Daltons.

Reference:

Vollmer, M. & van de Goor, T. HPLC-Chip/MS technology in proteomic profiling. Methods Mol Biol. **544**, 3–15, (2009).

B – Supporting Tables and Figures

Supplementary Table 1 and Tables 3-6. Raw CFG glycan microarray binding data for rBGL and each BGL mutant.

Note: Table 1 and Table 3-6 data is provided in an Excel spreadsheet that has been uploaded as a separate file. Each tab within the spreadsheet represents binding data for a separate BGL protein.

Supplementary Table 2. Mutants predicted to disrupt ligand binding in BGL.

Position	Original residue	Mutated residue	$\Delta\Delta G_{binding}$ score [1]
**Primary binding site (GalNAc)**			
107	ARG	TYR [2]	17.84
49	GLY	HIS [2]	16.59
49	GLY	ASN	6.40
71	HIS	GLU	6.21
72	ASN	LYS	6.10
48	SER	LYS	5.72
28	TYR	SER	5.71
49	GLY	LYS [3]	4.28
72	ASN	PRO [3]	4.09
48	SER	ASP [3]	3.68
**Secondary binding site (GlcNAc)**			
103	ARG	GLU [3]	17.24
103	ARG	TYR	12.63
78	ASP	ARG [3]	7.91
80	VAL	ASP	6.49
81	THR	ILE	5.60
81	THR	PRO	3.90
78	ASP	TRP [3]	2.73
116	VAL	ASP [3]	2.40
78	ASP	LYS [3]	2.17
114	TYR	TRP [4]	-10.93

[1] Mutants are ranked by $\Delta\Delta G_{binding}$ score, which is a difference between predicted MM-GBSA $\Delta G_{binding}$ scores of the mutant and the wild-type protein. A positive $\Delta\Delta G_{binding}$ value indicates that a mutant binds ligand in a respective binding site weaker than the wild-type protein. Mutants were predicted to disrupt binding in a respective binding site (see Materials and Methods for details); no change in binding was assumed for the alternative binding site.
[2] Mutant proteins did not show any expression.
[3] Mutant proteins were either insoluble or showed very low solubility.
[4] Y114W was used as a test mutation predicted to stabilize ligand binding.

Supplementary Table 7. O-glycan structures observed from fetuin. The numbers represent the relative abundance of each O-glycan structure.

**Glycans**	**Non-enriched**		**BGLwt**		**BGLmut**	
	**Biol. Rep. 1**	**Biol. Rep. 2**	**Biol. Rep. 1**	**Biol. Rep. 2**	**Biol. Rep. 1**	**Biol. Rep. 2**
HexNAc(1)		4.86	1.28	1.92	1.70	1.43
HexNAc(1)Hex(1)		0.88	1.32	0.87	1.58	1.83
HexNAc(1)Hex(1),HexNAc(1)Hex(1)					0.06	0.06
HexNAc(1)Hex(1)NeuAc(1)	9.67	25.04	31.56	32.35	49.48	55.73
HexNAc(1)Hex(1)NeuAc(2)	72.94	51.36	51.52	51.17	35.66	33.46
HexNAc(1)Hex(1)NeuAc(2),HexNAc(1)Hex(1)NeuAc(2)	0.15					
HexNAc(1)Hex(1)NeuGc(1)			0.42	1.54	1.64	1.26
HexNAc(1)Hex(1)NeuGc(1)NeuAc(1)	2.06	2.74	1.72	1.86	1.26	1.21
HexNAc(1)Hex(1)NeuGc(2)	8.20	5.53	5.15	5.00	3.49	3.47
HexNAc(2)Hex(1)NeuAc(1)					0.42	
HexNAc(2)Hex(2)NeuAc(1)	0.41	1.58	1.66	1.34	1.75	1.10
HexNAc(2)Hex(2)NeuAc(1),HexNAc(2)Hex(2)NeuAc(1)					0.06	
HexNAc(2)Hex(2)NeuAc(2)	6.49	8.03	5.39	3.95	2.90	0.46
HexNAc(2)Hex(2)NeuAc(2),HexNAc(2)Hex(2)NeuAc(2)	0.09					

Supplementary Table 8. Primers used in this study.

Primer	Sequence	Use
R107Y-F	AGCATACGCAtacGAGAAGCAACTCG	PCR
R107Y-R	CTCGGTCCATCATTGTAG	PCR
G49H-F	GGGTGGCAGCcacACCTCCGGCA	PCR
G49H-R	ATGGTGAGCACCTTCTCCC	PCR
G49N-F	GGGTGGCAGCaacACCTCCGGCA	PCR
G49N-R	ATGGTGAGCACCTTCTCCC	PCR
H71E-F	TTTGGGCGTTgaaAACAATAAGC	PCR
H71E-R	GCGACGATGAAGTCTTCG	PCR
N72K-F	GGGCGTTCACaaaAATAAGCGGT	PCR
N72K-R	AAAGCGACGATGAAGTCTTCG	PCR
S48K-F	CATGGGTGGCaaaGGAACCTCCGG	PCR
S48K-R	GTGAGCACCTTCTCCCCG	PCR
Y28S-F	CGTCTGGAACtccGCCAATGGTG	PCR
Y28S-R	GTCTGCTCAACGATCCTG	PCR
G49K-F	GGGTGGCAGCaaaACCTCCGGCA	PCR
G49K-R	ATGGTGAGCACCTTCTCCC	PCR
N72P-F	GGGCGTTCACccgAATAAGCGGTG	PCR
N72P-R	AAAGCGACGATGAAGTCTTC	PCR
S48D-F	CATGGGTGGCgacGGAACCTCCG	PCR
S48D-R	GTGAGCACCTTCTCCCCG	PCR
R103E-F	TGATGGACCGgaaGCATACGCAAG	PCR
R103E-R	TTGTAGTACTGGGGGTTG	PCR
R103Y-F	TGATGGACCGtacGCATACGCAAG	PCR
R103Y-R	TTGTAGTACTGGGGGTTG	PCR
D78R-F	GCGGTGGTGCcgcATCGTCACGA	PCR
D78R-R	TTATTGTTGTGAACGCCCAAAG	PCR
V80D-F	GTGCGACATCgacACGAGCCTCG	PCR
V80DR	CACCGCTTATTGTTGTGAACGC	PCR
V80D-F	GTGCGACATCgacACGAGCCTCG	PCR
V80D-R	CACCGCTTATTGTTGTGAACGC	PCR
T81I-F	CGACATCGTCatcAGCCTCGCTG	PCR
T81I-R	CACCACCGCTTATTGTTG	PCR
T81P-F	CGACATCGTCccgAGCCTCGCTG	PCR
T81P-R	CACCACCGCTTATTGTTGTGAAC	PCR
D78W-F	GCGGTGGTGCtggATCGTCACGAG	PCR

D78W-R	TTATTGTTGTGAACGCCC	PCR
V116D-F	GACCTACAACgacGGCAATGCGC	PCR
V116D-R	GCGAGTTGCTTCTCTCTTGC	PCR
D78K-F	GCGGTGGTGCaaaATCGTCACGA	PCR
D78K-R	TTATTGTTGTGAACGCCCAAAG	PCR
Y114W-F	ACTCGCGACCtggAACGTCGGCA	PCR
Y114W-R	TGCTTCTCTCTTGCGTATGC	PCR
BGL-F	TTTAAGAAGGAGATATACATATGAGCTACAAAATCT GCG	Cloning
BGL-R	CAGTGGTGGTGGTGGTGGTGCTCGAGTTATCAT CCGATGATTATGTTG	Cloning
rBGL-F	GATATACATATGAGCTACAAAATCTGCGTC	Cloning
rBGL-R	CTCGAGTTATCATCCGATGATTATGTTGGCCTT	Cloning
ITS1-F	CTTGGTCATTTAGAGGAAGTAA	PCR
ITS4	TCCTCCGCTTATTGATATGC	PCR

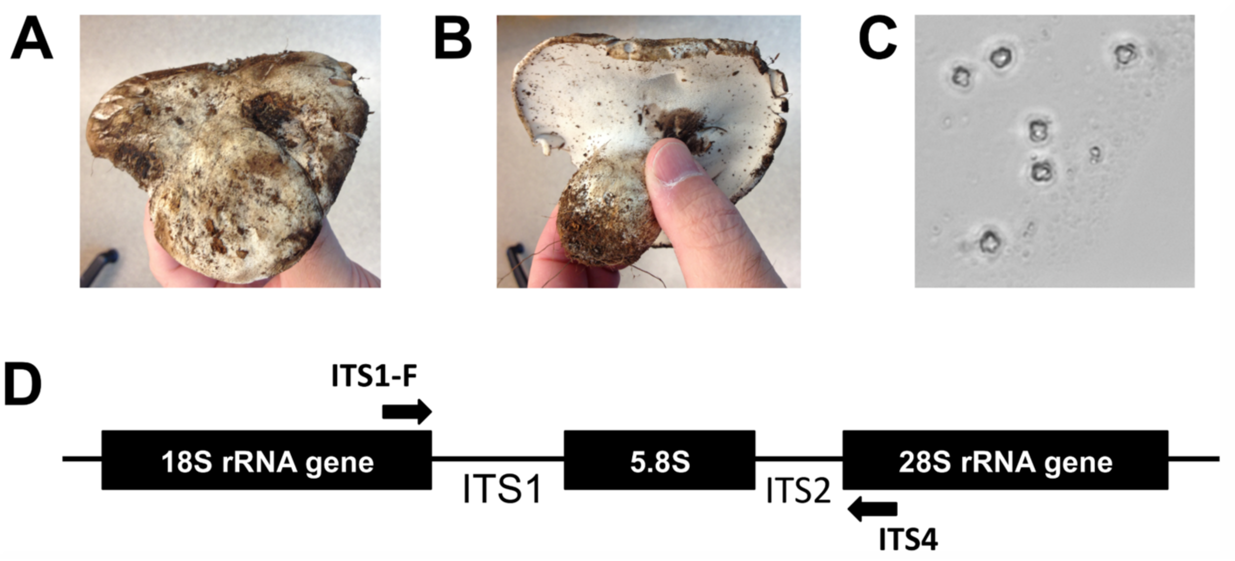

Figure S1. Morphological and molecular mushroom speciation. The sourced mushroom had dark gray and brown caps **(A and B)** and produced a spore print **(C)** of small elliptical bumpy spores. **(D)** A schematic depicting the fungal internal transcribed spacers (ITS). The regions that lie between the 18S ribosomal RNA gene (rRNA) and 5.8S rRNA gene (ITS1) and between the 5.8S rRNA gene and 28 rRNA gene (ITS2) were amplified using primers ITS1-F and ITS4 primers.

```
Sourced mushroom    -ACCTGCGGAAGGATCATTAATGAAATGAAGCATGTGAAGGGTTGTAGCTGGTCTCTTTT 59
B. grisea           -ACCTGCGGAAGGATCATTAATGAAATGAAGCATGTGAAGGGTTGTAGCTGGTCTCTTTT 59
B. leucomelaena     AACCTGCGGAAGGATCATTAATGAAATGAAGCATGTGAAGGGTTGTAGCTGGCCTCTTTT 60

Sourced mushroom    TTGGAGGCATGTGCACGCCCGGATCATTCATCCCCTTCACACACCTGTGCACAACCTGTA 119
B. grisea           TTGGAGGCATGTGCACGCCCGGATCATTCATCCCCTTCACACACCTGTGCACAACCTGTA 119
B. leucomelaena     CTGGAGGCATGTGCACGCCTGGATCATTCATCCCCTTCACACACCTGTGCACAACCTGTA 120

Sourced mushroom    GCTTGGGATGATCACGGAGGCCATCTTTTTTTGGTGGCGCCGAATGCCCTTGCTATGATC 179
B. grisea           GCTTGGGATGATCACGGAGGCCATCTTTTTTTGGTGGCGCCGAATGCCCTTGCTATGATC 179
B. leucomelaena     GCTTGGGATGATCACGGAGGCTGTCTTTC--TGGTGGCACCGAATGCCCTTGCTATGATC 178

Sourced mushroom    TTTTGATACACACCTTTATAACGTTTATGTTGATGACATTATTTTGAACGGTAATACAAC 239
B. grisea           TTTTGATACACACCTTTATAACGTTTATGTTGATGACATTATTTTGAACGGTAATACAAC 239
B. leucomelaena     TTTTGATACACACCTTTATAACGTTCATGTCGACGATATTATTTTGAATGTAAATACAAC 238

Sourced mushroom    TTTCAGCAACGGATCTCTTGGCTCTCGCATCGATGAAGAACGCAGCGAAATGCGATAAGT 299
B. grisea           TTTCAGCAACGGATCTCTTGGCTCTCGCATCGATGAAGAACGCAGCGAAATGCGATAAGT 299
B. leucomelaena     TTTCAGCAACGGATCTCTTGGCTCTCGCATCGATGAAGAACGCAGCGAAATGCGATAAGT 298

Sourced mushroom    AATGTGAATTGCAGAATTCAGTGAATCATCGAATCTTTGAACGCACCTTGCACTCCTTGG 359
B. grisea           AATGTGAATTGCAGAATTCAGTGAATCATCGAATCTTTGAACGCACCTTGCACTCCTTGG 359
B. leucomelaena     AATGTGAATTGCAGAATTCAGTGAATCATCGAATCTTTGAACGCACCTTGCACTCCTTGG 358

Sourced mushroom    TATTCCGAGGAGTATGCCTGTTTGAGCGTCATGAAATTCTCAACTGCCTTGGCCTTTTGT 419
B. grisea           TATTCCGAGGAGTATGCCTGTTTGAGCGTCATGAAATTCTCAACTGCCTTGGCCTTTTGT 419
B. leucomelaena     TATTCCGAGGAGTATGCCTGTTTGAGTGTCATGAAATTCTCAACTGCCTTGGCCTTTTGT 418

Sourced mushroom    GCCAAAGTGAAGTTGGATTTGGAGGTTTTGTTGCTGGCATGGAGCGTCTGGTTGATGCTT 479
B. grisea           GCCAAAGTGAAGTTGGATTTGGAGGTTTTGTTGCTGGCATGGAGCGTCTGGTTGATGCTT 479
B. leucomelaena     GCCAAAGTGAAGTTGGATTTGGAGGTTTTGTTGCTGGCATGGAGCGTCTGATTGATGCTT 478

Sourced mushroom    GCT-TGTTGGCTCCTCCTAAATGCATGCAGAGCTTGGCAAAGCGGCGTCGATGTGATAAT 538
B. grisea           GCT-TGTTGGCTCCTCCTAAATGCATGCAGAGCTTGGCAAAGCGGCGTCGATGTGATAAT 538
B. leucomelaena     GCTGTGTTGGCTCCTCCTAAAAGCATGCAGAGCTTGGCAAAGCGGCGTCCATGTGATAAT 538

Sourced mushroom    TATCTACATTGTCGTCAAATTGTGCAAATCTGCTGTGGGGGTTGCGAC-TTTATGAACAA 597
B. grisea           TATCTACATTGTCGTCAAATTGTGCAAATCTGCTGTGGGGGTTGCGAC-TTTATGAACAA 597
B. leucomelaena     TATCTACGTTGTCGTCGAAGTGTGCAGATCTGCTGTGGGG--TGTGACCTTTTTGAACAA 596

Sourced mushroom    TTTGACCTCAAATCAGGTAGGACTACCCGCTGA-------------------------- 630
B. grisea           TTTGACCTCAAATCAGGTAGGACTACCCGCTGAACTTAAGCATATCAATAAGCGGAGGA 656
B. leucomelaena     TTTGACCTCAAATCAGGTAGGACTACCCGCTGAACTTAAGCATATCAATAAGCGGAGGA 655
```

Figure S2. Nucleic acid sequence alignment of the ITS region of *Boletopsis* mushrooms. ClustalW was used to align DNA sequences of the ITS amplicon from the sourced mushroom (as described in Figure S1) and sequences of the same region from *B. grisea* (Accession No. EF457902) and *B. leucomelaena* (Accession No. DQ408771) present in GenBank. ITS1 and ITS2 are depicted with green and yellow highlights, respectively. The ITS sequences of the sourced mushroom and *B. grisea* are identical. Sequence positions in ITS1 and ITS2 that are different between the sourced mushroom and *B. leucomelaena* are shown indicated by blue highlight. Gaps introduced by ClustalW to yield the best alignment are depicted with hyphens.

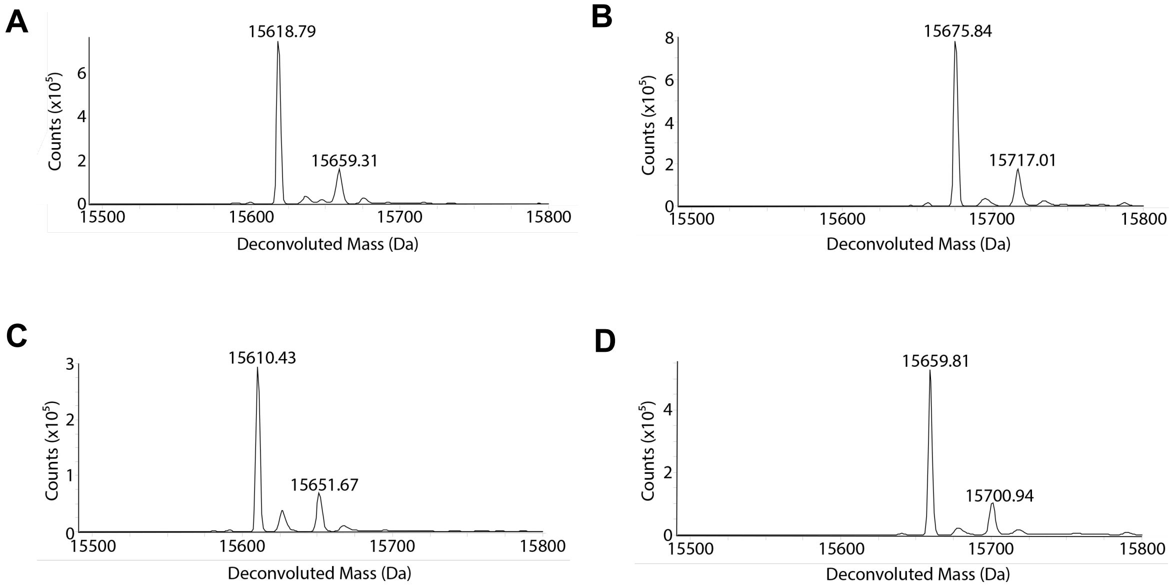

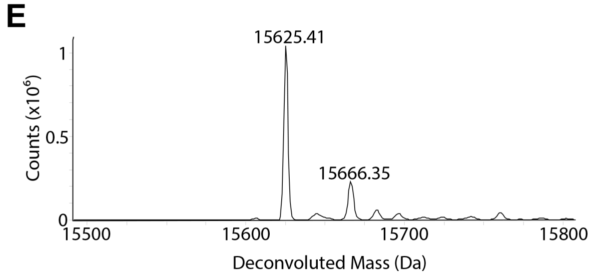

F

	Calculated MW (minus 1st Met) (Da)	TOF (intact mass) (Da)
rBGL-WT	15618.33	15618.79
rBGL-G49N	15675.38	15675.84
rBGL-H71E	15610.30	15610.43
rBGL-S48K	15659.42	15659.81
rBGL-R103Y	15625.32	15625.41

Figure S3. Acquired mass spectra of rBGL and mutants. Samples were analyzed by reverse phase liquid chromatography (LC) and electrospray ionization time-of-flight mass spectrometry (ESI-TOF MS). **(A)** recombinant BGL; **(B)** G49N mutant; **(C)** H71E mutant; **(D)** S48K mutant; **(E)** R103Y mutant; The additional peaks are within range of common modifications such as oxidation and acetylation. **(F)** The intact mass obtained for each protein corresponded to the calculated molecular weight lacking the initiating methionine using the ExPASy Compute pI/Mw tool (https://web.expasy.org/compute_pi/).

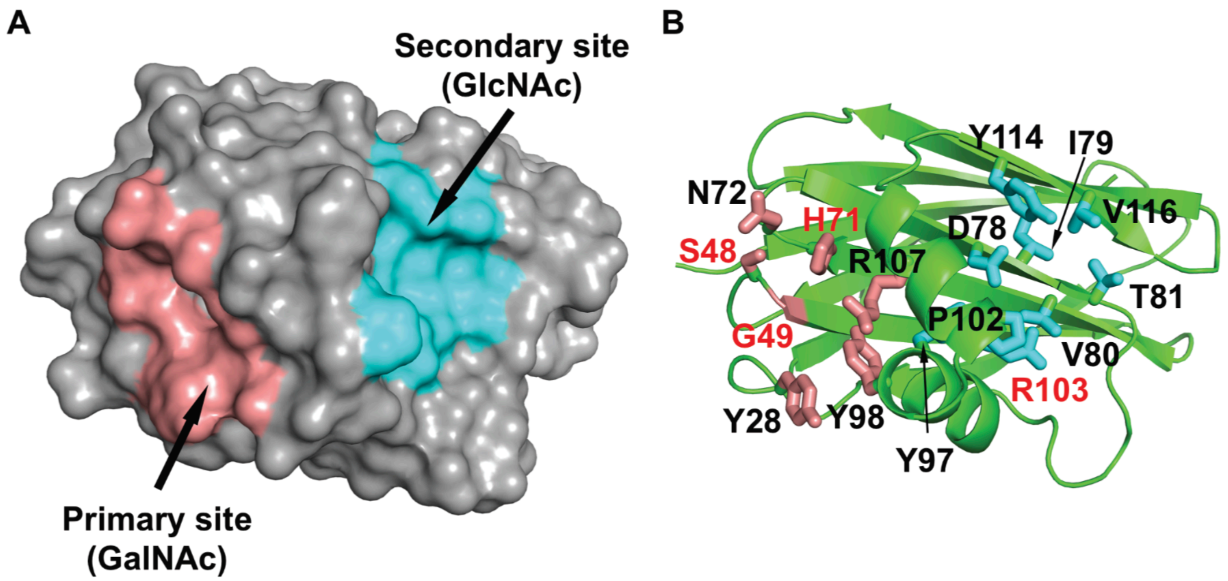

Figure S4. The overall structural model of rBGL. (A) Primary and secondary ligand binding sites are located on the same side of the protein. **(B)** Seven and nine residues comprise the binding pockets for GalNAc and GlcNAc monosaccharides, respectively. Four amino acids (in red) were individually mutated to disrupt binding in the respective pockets. These positions are shown in greater detail in Figure 3 of the main text. Molecular graphic images were generated using PyMOL software (www.pymol.org) by V. Potapov.

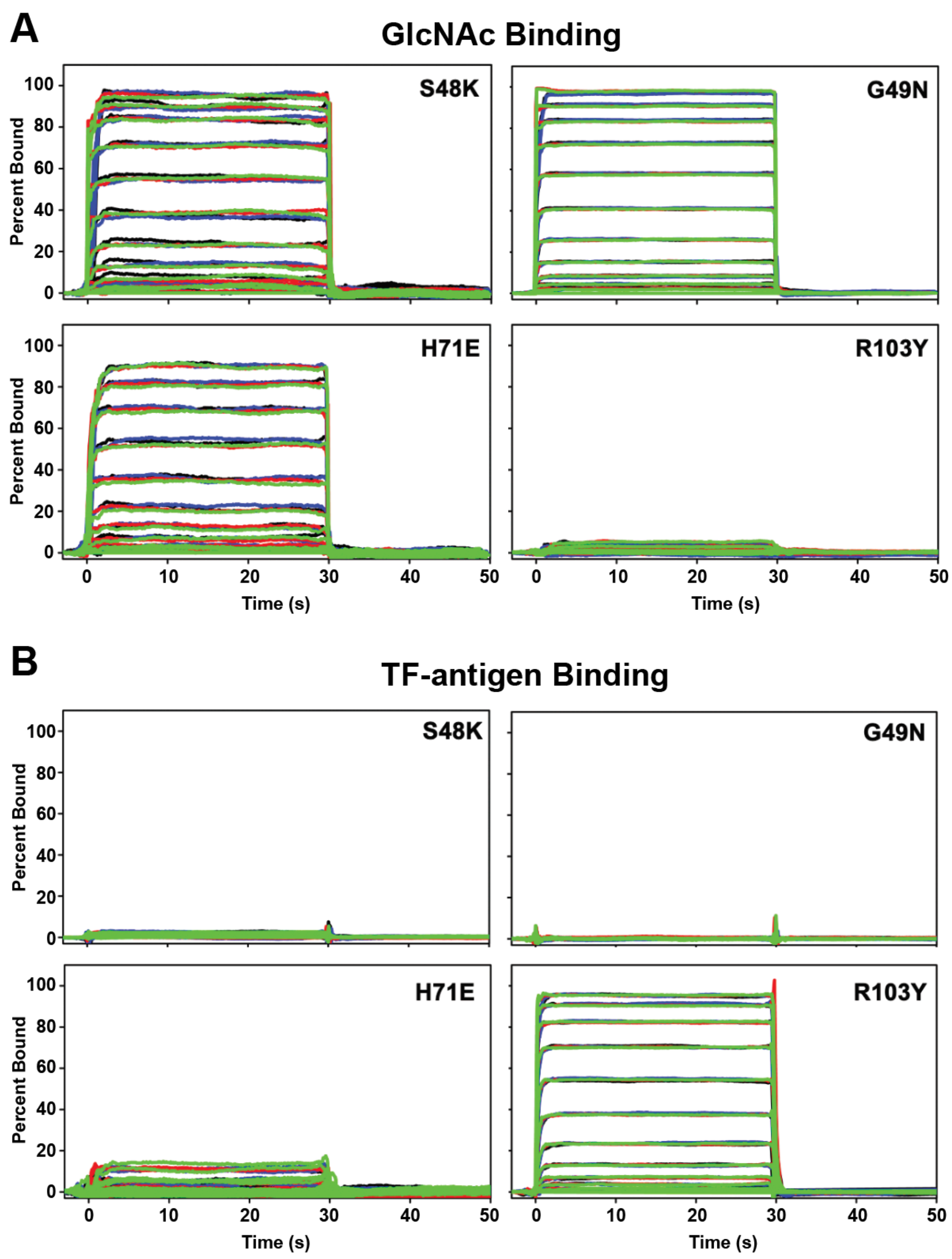

Figure S5. SPR binding analysis of BGL mutants. (A) GlcNAc and **(B)** TF-antigen were tested for binding to immobilized BGL mutants (S48K, G49N, H71E, and R103Y) using a 2-fold concentration series up to 10 mM and 2 mM, respectively. Shown are sensorgrams captured for 8 replicate binding experiments (overlaid colored lines). For all experiments, equilibrium curves for binding data are shown in Supplemental Figure S6.

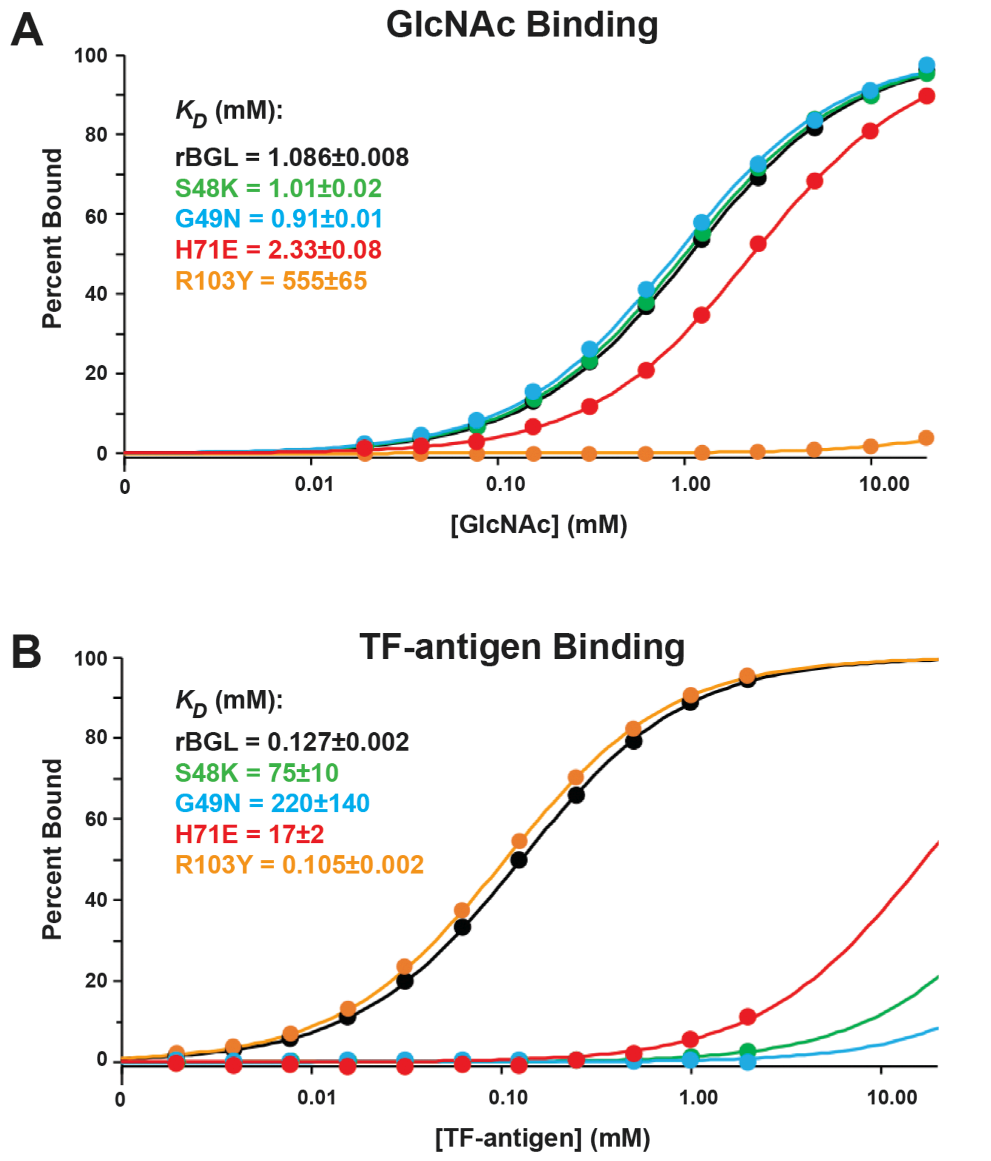

Figure S6. SPR equilibrium data of BGL mutants. For **(A)** GlcNAc and **(B)** TF-antigen binding data presented in Supplemental Figure S9, equilibrium dissociation binding constants were determined by fitting the data at equilibrium to a 1:1 interaction model using the software Scrubber 2.

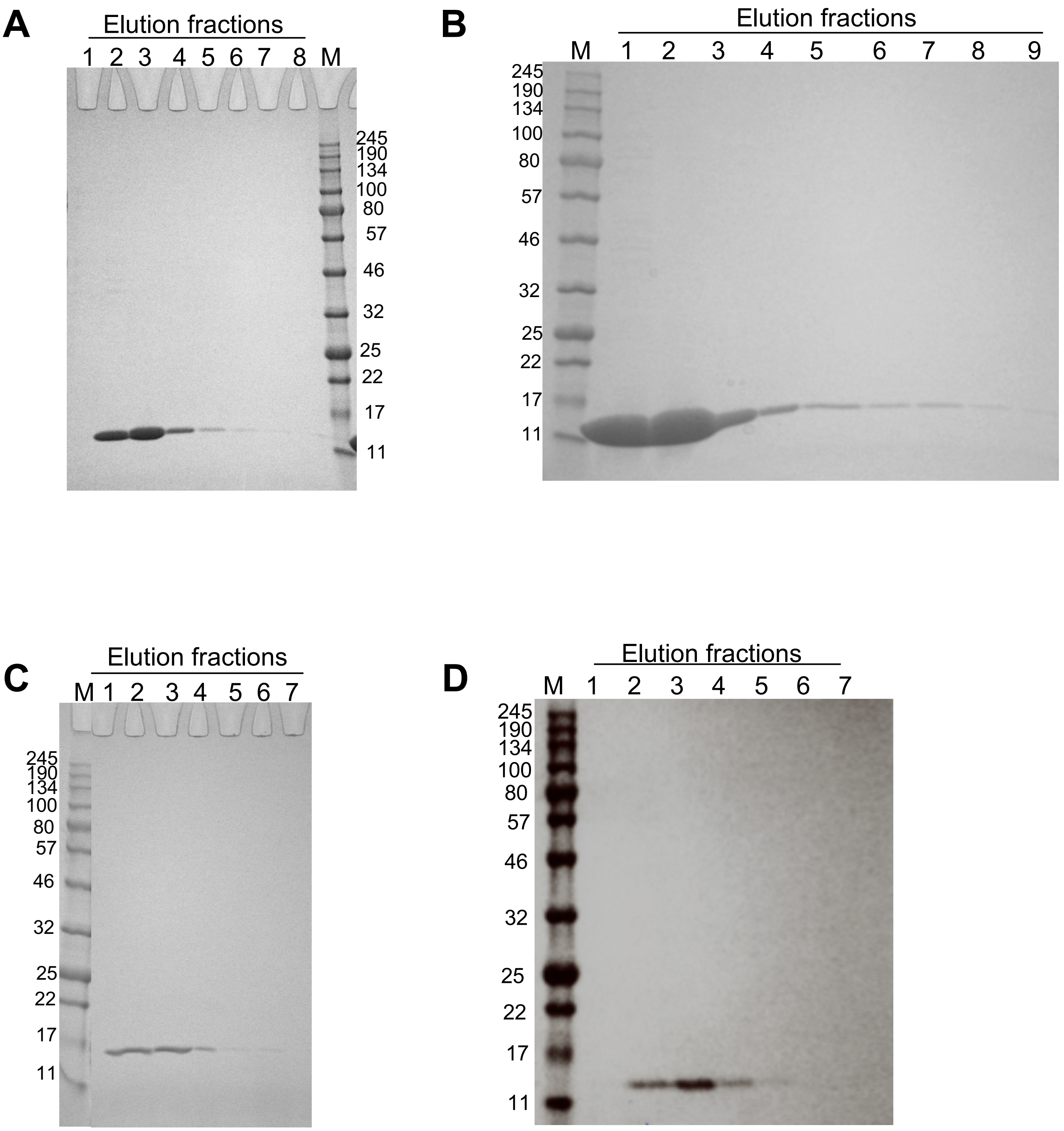

Figure S7. Purification of recombinant BGL mutants. SDS-PAGE of the elution fractions from large scale purifications (1-6 L cultures) are shown. Mutants G49N **(A)**, H71E **(B)** and S48K **(C)** were each passed over a GlcNAc-agarose column while R103Y **(D)** was purified using a Galβ1,3GalNAc-agarose column. M, a broad range pre-stained protein standard (kDa, New England Biolabs #P7712S).

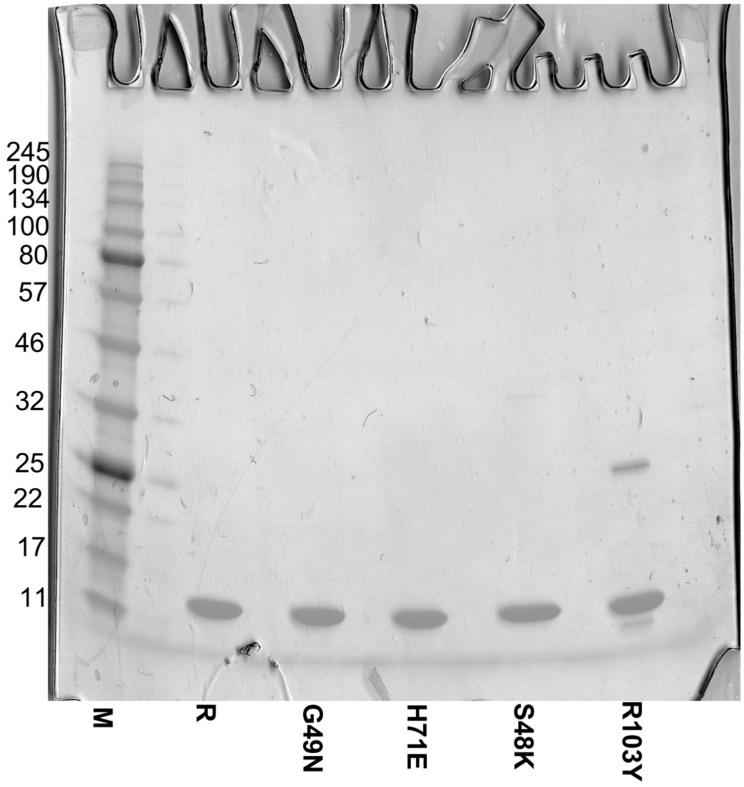

Figure S8. Purified proteins used for SPR analysis. Shown is the uncropped gel image of the purified proteins used for surface plasmon resonance (SPR). Protein concentration was determined by Bradford assay and 5 μg of each protein was separated by SDS-PAGE. Recombinant BGL (R) and mutants G49N, H71E and S48K were purified by affinity chromatography using GlcNAc agarose (as described in Materials and Methods in the main text), and mutant R103Y was purified using Galβ1,3GalNAc-agarose. M, broad range pre-stained protein standard (kDa, New England Biolabs #P7712S).

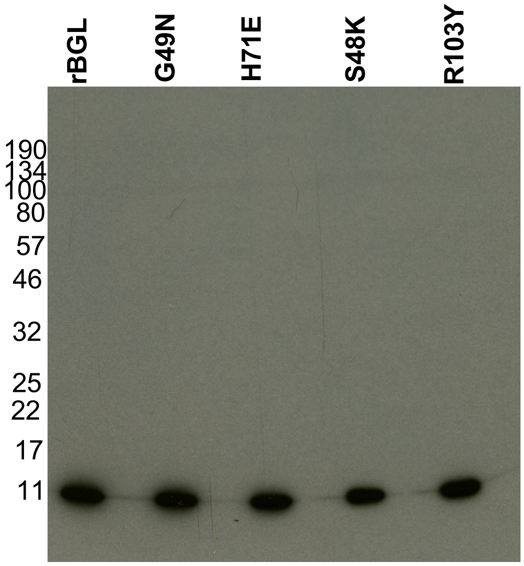

Figure S9. Western blot analysis of purified proteins. Shown is a Western blot of the purified BGL proteins. Protein concentration was determined by Bradford assay and 100 ng of each protein was separated by SDS-PAGE and transferred to nitrocellulose membrane. A western blot assay was performed using an anti-BGL antibody (1:1000 in 3% non-fat dry milk/PBS-T) followed by anti-rabbit HRP linked (1:2000 in 3% non-fat dry milk/ PBS-T). Numbers reflect the size (kDa) and position of the proteins from a broad range pre-stained protein standard (New England Biolabs #P7712S).

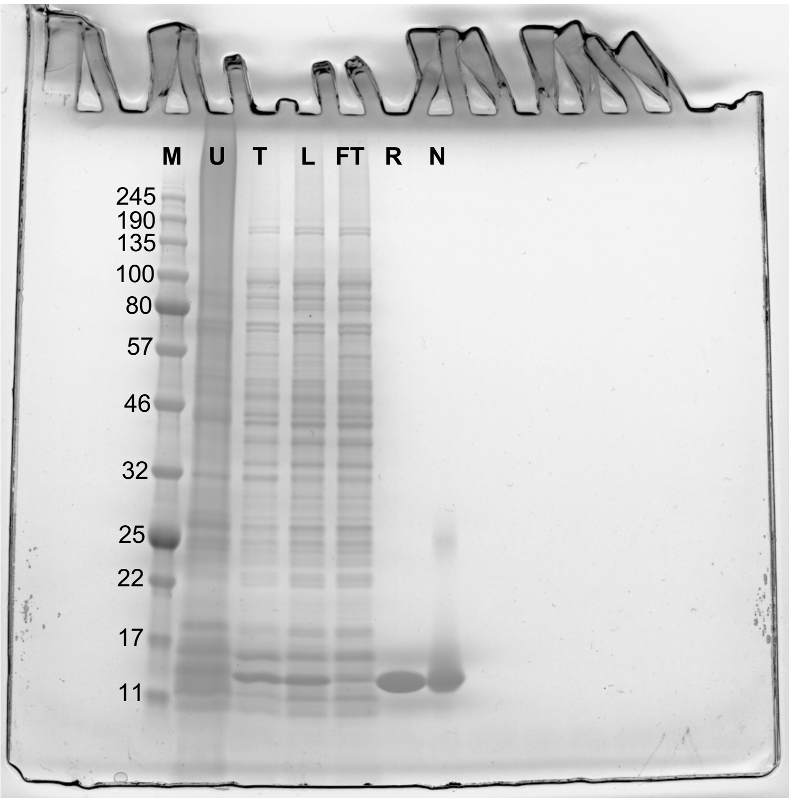

Figure S10. SDS-PAGE of *E. coli* expressed and purified recombinant BGL. Shown here is the uncropped gel image presented in Figure 1 of the main text. *E. coli*-expressed and purified recombinant BGL was visualized by separation via 10-20% Tris-glycine SDS-PAGE and SimplyBlue staining. M: M, broad range pre-stained protein standard (kDa, New England Biolabs #P7712S); U: uninduced lysate; T: total induced lysate; L: soluble lysate; FT: column flow-through; R: purified recombinant BGL; N: purified native BGL.

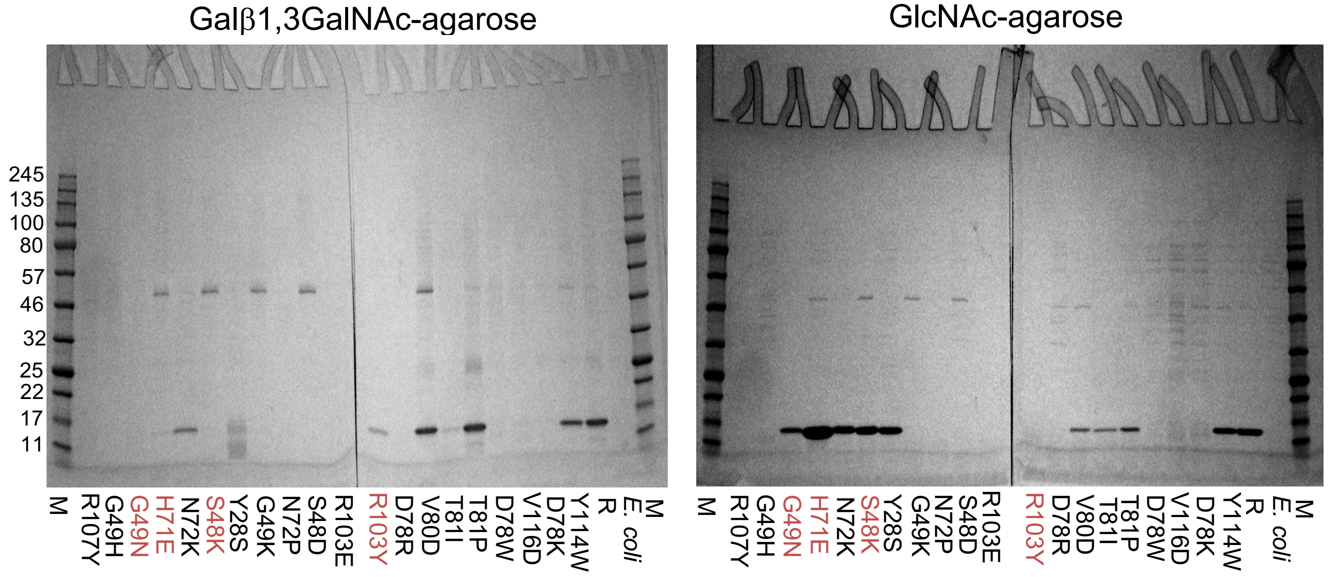

Figure S11. Qualitative assessment of mutant lectin specificity. Shown here are the uncropped gel images presented in Figure 3 of the main text. Computationally predicted mutants that disrupt each binding site were created by site-directed mutagenesis and expressed in *E. coli*. Aliquots of each lysate were separately passed over Galβ1,3GalNAc-agarose and GlcNAc-agarose columns. Proteins that bound to each column were eluted and separated by SDS-PAGE. Mutants G49N, H71E and S48K bound to GlcNAc but not Galβ1,3GalNAc-agarose. Mutant R103Y bound to Galβ1,3GalNAc but not GlcNAc-agarose. Recombinant BGL (R) and *E. coli* extract were used as controls. Orange text denotes mutants with differential binding ability. M, broad range pre-stained protein standard (kDa, New England Biolabs #P7712S).
